# The extracellular domain of *Sa*NSrFP binds bacitracin and allows the identification of new members of the BceAB transporter family

**DOI:** 10.3389/fmicb.2025.1662803

**Published:** 2025-09-17

**Authors:** Christian Mammen, Julia Gottstein, Pablo Cea, Kira Tantsur, Jens Reiners, Michele Bonus, Holger Gohlke, Sander H. J. Smits

**Affiliations:** ^1^Institute of Biochemistry, Heinrich Heine University Düsseldorf, Düsseldorf, Germany; ^2^Institute for Pharmaceutical and Medicinal Chemistry, Heinrich-Heine-University Düsseldorf, Düsseldorf, Germany; ^3^Center for Structural Studies, Heinrich Heine University Düsseldorf, Düsseldorf, Germany; ^4^Institute of Bio- and Geosciences (IBG-4: Bioinformatics), Forschungszentrum Jülich, Jülich, Germany

**Keywords:** BceAB, antimicrobial resistance, lantibiotic, ABC transporter, human pathogen

## Abstract

Peptidoglycan serves as the first permeability barrier of Gram-positive bacteria. Intermediates of the peptidoglycan synthesis cycle are typical targets of antimicrobial compounds, including the peptide antibiotics nisin and bacitracin. In human pathogenic bacteria, gene clusters have been identified that are upregulated to confer resistance against these compounds. One such cluster found in *Streptococcus agalactiae* encodes a Bacitracin efflux (BceAB)-type ATP binding cassette transporter, *Sa*NsrFP, and an associated two-component system, *Sa*NsrRK. *Sa*NsrFP has been shown to confer resistance against multiple antimicrobial peptides *in vivo*, with highest activity against bacitracin. Like other BceAB-type ABC-transporters, *Sa*NsrFP features a large extracellular domain (ECD) that determines the substrate spectrum. Here, we report the purification and *in vitro* characterization of the *Sa*NsrFP complex. Measuring the ATPase activity in the presence and absence of bacitracin showed that the binding of bacitracin allosterically modulates ATPase activity. By expressing and purifying only the soluble ECD of *Sa*NsrP, we could show through two *in vitro* binding assays that this segment alone is responsible for bacitracin binding and then explored the putative binding mechanism using molecular docking. Additionally, we assessed the structural conservation of the ECD across 24 BceAB-type ABC-transporters with the AlphaFold database. Enabling us to create a first classification within this superfamily based on the structural fold of the ECD.

## 1 Introduction

Adaptation is one of the key factors that enable bacteria to conquer and thrive in a rapidly changing environment. As the cell wall is the first point of contact, its maintenance, modification, and protection are crucial to shield bacteria from harmful environmental factors, such as ions, pH, or toxic compounds. Peptidoglycan constitutes the first permeability barrier in Gram-positive bacteria, and its synthesis relies on lipid II. Therefore, the lipid II cycle is a vital process and target for many antimicrobial compounds. Inhibiting this cycle leads to cells incapable of extending the peptidoglycan layer, inhibits cell division, and subsequently leads to cell death. Thus, intermediates of the lipid II cycle are well-studied targets for antibiotics such as bacitracin, nisin, or vancomycin (Muller et al., 2017). To survive antimicrobial exposure, the bacterial cell needs to accurately assess the severity of the situation to react with a minimized metabolic cost. Here, the presence of antimicrobials is sensed by two-component systems (TCS), which induce the expression of resistance proteins resulting in different adaptive responses, for example, modulation of the membrane composition (Fritz et al., 2015, Tollerson and Ibba, 2020).

Moreover, mainly in soil and human pathogenic Gram-positive bacteria, gene clusters were identified encoding for a resistance system containing membrane-embedded proteins, such as the Bacitracin efflux (BceAB)-type ATP-binding cassette (ABC)-transporter family (Orlando, 2024; Dintner et al., 2011).

**Figure 1:**
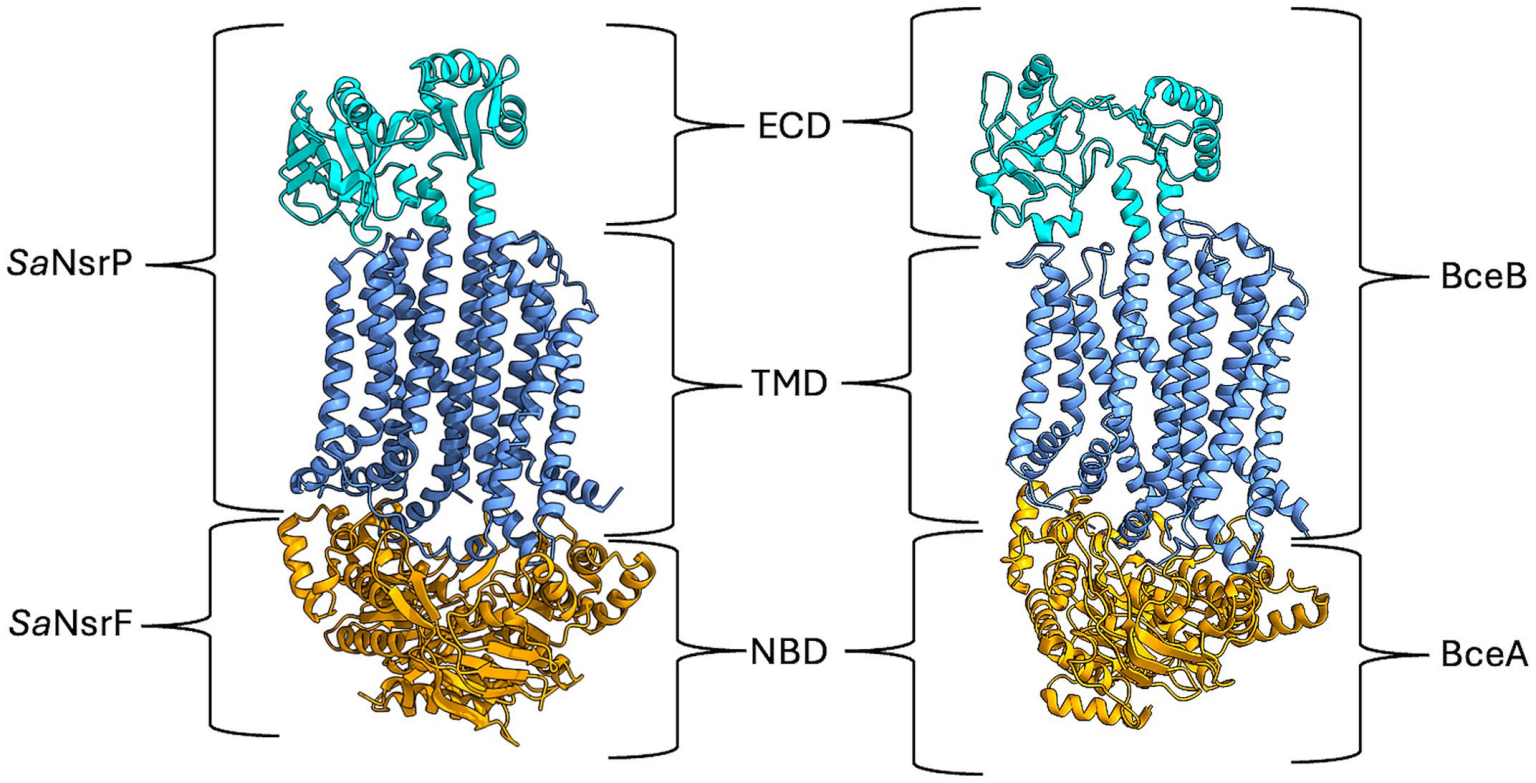
AlphaFold model of SaNsrFP and cryo-EM structure of BceAB from *B. subtilis* [7TCG, (George et al., 2022)]. The ECD of SaNsrP and BceB is highlighted in cyan, while the TMD is depicted in cornflower blue. Lastly, the dimeric NBD SaNsrF and BceA are marked in orange. Image was created with ChimeraX version 1.8.

Recently, the cryo-electron microscopy structure of BceAB from *B. subtilis* was solved (George et al., 2022; George and Orlando, 2023) and revealed that this transporter consists of two nucleotide-binding domains, which hydrolyze ATP to generate energy, and a single transmembrane domain, consisting of 10 transmembrane helices (TMH). TMH 1 to 4 and TMH 7 to 10 form bundles that are related by two-fold pseudosymmetry, representing an FtsX-domain fold similar to type VII ABC transporters involved in mechanotransmission (Thomas et al., 2020). The overall arrangement of the TMHs of BceB is asymmetrical due to the closer position of TMH 5 and 6 to TMH 7 to 10 than to the other TMH bundle (George et al., 2022). TMH 7 and 8 form longer and extended stalk helices with a 200–250 amino acid large extracellular domain (ECD) in between, which is the hallmark of BceAB-type transporters (Figure 1) (Khosa et al., 2013). Since BceAB confers resistance against bacitracin, it is proposed that it detects the complex of bacitracin with undecaprenylpyrosphate (UPP) (Kobras et al., 2020). In the structure of BceAB, between TMH 5, 6 and TMH 7, 9, a hydrophobic lipid-binding pocket with a suggested bound UPP derivate 4-amino-4-deoxy-L-arabinopyranosyl undecaprenyl phosphate (AUP) was identified, which is situated directly underneath the ECD (George et al., 2022). It is proposed that native UPP and other UPP-lipid derivates might bind to this binding site. Mutation studies replacing the ECD of VraG in *S. aureus* with its counterpart from VraE, responsible for bacitracin resistance, led to enhanced bacitracin resistance in VraG and increased sensitivity to colistin (Falord et al., 2012; Hiron et al., 2011; Cho et al., 2022). This suggests that the ECD is involved in substrate sensing, particularly, that it binds bacitracin when bound to UPP (Kobras et al., 2020).

Within most *bce* operons, a co-evolved BceRS-type TCS is present consisting of a response regulator (BceR) and a transmembrane histidine kinase (HK, BceS) sensor. In contrast to other HK proteins, BceS has a notoriously short extracellular domain of roughly 25 amino acids, which is likely too small to allow an effective binding to the target lantibiotic (Rietkötter et al., 2008; Mascher et al., 2003; Mascher, 2006; Clemens et al., 2017; Khosa et al., 2013). Thus, the currently proposed signal transduction pathway is that BceAB senses the presence of an antimicrobial peptide and activates HK through contacts between the transmembrane regions (Gebhard, 2012; Revilla-Guarinos et al., 2014). This process was described for the detoxification system GraRS-VraFG in *S. aureus* (Cho et al., 2021) and various TCS-ABC transporters in *B. subtilis* (BceRS-AB, YxdJK-LM, and YvcPQ-RS) (Dintner et al., 2011; Fritz et al., 2015). Furthermore, it has been shown that the antimicrobial activity of the transporter depends on ATP hydrolysis through the ATPase BceA (Rietkötter et al., 2008) as well as the complex formation with BceRS (Dintner et al., 2014). Major efforts have been made to unravel the mechanism of BceAB-type transporters. Proposals ranged from AMP removal from the membrane (Gebhard and Mascher, 2011) over functioning as an exporter (Reiners et al., 2017) to flipping the UPP (Kingston et al., 2014). A more recent study postulated a target-AMP-dissociative, ATP hydrolysis-driven mechanism for BceAB-type transporters, in which the target-AMP complex is recognized and UPP is physically released from the grip of bacitracin (Kobras et al., 2020).

Recently, a high level of resistance against bacitracin was observed for the BceAB transporter from *S. agalactiae* COH1, *Sa*NsrFP, which was previously found to also mediate resistance against nisin, an antimicrobial peptide belonging to the lantibiotic family (Gottstein et al., 2022; Reiners et al., 2020) even in the absence of the TCS. *Sa*NsrFP consists of a homodimeric nucleotide-binding domain, NsrF, that can hydrolyze ATP and the monomeric transmembrane protein NsrP. It was shown that the observed resistances mediated by *Sa*NsrFP are ATP hydrolysis-dependent by using an ATP hydrolysis-deficient mutant of *Sa*NsrFP, termed *Sa*NsrF_H202A_P (Furtmann et al., 2020; Gottstein et al., 2022).

To analyze the interplay between the ECD and its substrate bacitracin, we investigated the influence of substrate binding on the *in vitro* ATPase activity of *Sa*NsrFP. We further set out to measure the binding affinity of bacitracin and Zn-bacitracin towards the purified ECD and identified a putative binding mode for bacitracin through molecular docking experiments. To translate our findings to other BceAB-type ABC-transporters, we searched for and identified new members of this family using structural homology and could classify them by the distinct architecture of the ECD into five distinct groups.

## 2 Materials and methods

### 2.1 Heterologous expression and purification of *Sa*NsrFP in *E. coli* (DE3) C41 dd

SaNsrFP (Uniprot ID: X5KGL2 + Q8DZX0) was cloned from pIL-sv-NsrFP into pET16b for heterologous expression of *Sa*NsrFP with an N-terminal 10x-His-tag at *Sa*NsrF in *E. coli* (DE3) C41 dd cells (Kanonenberg et al., 2019). Expression was performed at 18 °C overnight in LB. Resuspended cells were lysed at 1.5 kbar with a cell disruptor (Microfluidics) and membranes were collected after two centrifugation steps at 20,000 × *g* and 150,000 × *g*, respectively. Membranes were homogenized with resuspension buffer and stored at −80 °C. For purification, membranes were solubilized with 1% (w/V) LMNG and subjected to IMAC followed by SEC. The detailed procedures are described in the Supplementary material.

### 2.2 ATPase activity measurements of *Sa*NsrFP in LMNG micelles

Purified *Sa*NsrFP was diluted in purification buffer (100 mM NaCl, 50 mM HEPES pH 8) supplemented with 0.005% (w/V) LMNG to 0.1 mg/mL. For the ATPase kinetic measurements, ATP stock concentrations ranging from 0.5–50 mM were prepared. For each sample, 21 μL *Sa*NsrFP (2.1 μg per sample) was supplemented with 3 μL 100 mM MgCL_2_ (final concentration 10 mM) or bidest H_2_O (background measurement) and subsequently, the reaction was started with 6 μL of the respective ATP stock. Reaction mixes were incubated at 30 °C for 30 min. A 96-well plate featuring a phosphate standard ranging from 0 to 12.5 nmol phosphate was prepared with 175 μL 40 mM H_2_SO_4_. To stop the reaction, 25 μL of the reaction mix was added to the H_2_SO_4_ solution. Next, the solution was stained with staining solution (15.7% H_2_SO_4_, 0.1% Malachite green, 1.5% Ammonium molybdate, 0.17% Tween 20) for 8 min at room temperature. Afterwards, the absorbance of each well was measured at 595 nm and the resulting values were analyzed with GraphPad Prism version 10.3.1 for MacOS, GraphPad Software, San Diego, California USA, www.graphpad.com and the Michaelis Menten fit. For the ATPase kinetics containing 0.1 mM bacitracin, *Sa*NsrFP was spiked with either 10 mM MgCl_2_ and 0.1 mM bacitracin or bidest H_2_O and 0.1 mM bacitracin. All positive and background measurements were done in at least duplicates.

### 2.3 Heterologous expression and purification of *Sa*NsrP-ECD in *E. coli* (DE3) BL21

*Sa*NsrP-ECD (aminoacids 311 to 512) was cloned from pIl-sv-NsrFP vector into pET28b with an N-terminal 6xHis tag. *Sa*NsrP-ECD was expressed in *E. coli* (DE3) BL21 in LB supplemented with 30 μg/mL kanamycin overnight at 18 °CAfter overnight expression, cells were harvested at 5,000 × *g* for 15 min at 4 °C and resuspended in resuspension buffer (50 mM Tris pH 8.0, 50 mM NaCl, 10% Glycerol) supplemented with 1,000 U of DNAse, RNAse and one protease inhibitor tablet (Roche). After cell disruption at 1.5 kbar, cell debris and membranes were removed by ultracentrifugation at 150,000 × *g* for 1 h at 4 °C. The supernatant was purified by IMAC, followed by thrombin cleavage, reverse IMAC and SEC. Detailed procedures are stated in the Supplementary material.

### 2.4 Multi-angle light scattering (MALS)

Purified ECD was concentrated to 2 mg/mL using centrifugal filters with a 3-kDa cut-off (Amicon Ultra-0.5 MERCK/ Millipore) and the samples were centrifuged at 100,000 × *g* at 4 °C for 30 min. For the measurement with Zn-bacitracin, the protein sample was preincubated with 1 mM Zn-bacitracin. Superdex 75 Increase 10/300 GL column (GE Healthcare) was pre-equilibrated overnight at 0.1 mL/min flow rate with buffer (20 mM Tris pH 8.0, 500 mM NaCl). For each analysis, 200 μL of a protein sample at 2.0 mg/mL concentration was loaded onto the column at 0.6 mL/min flow rate using a 1,260 binary pump (Agilent Technologies). The scattered light was measured with a miniDAWN TREOS II light scatterer, (Wyatt Technologies), and the refractive index was measured with an Optilab T-rEX refractometer (Wyatt Technologies). Data analysis was performed with ASTRA 7.3.2.21 (Wyatt Technologies) (Slotboom et al., 2008).

### 2.5 Tyrosine fluorescence quenching

The measurements were conducted using a Fluorolog Jobin Yvon FL-3-11. 1 mL of NsrP-ECD solution in SEC buffer (25 mM Tris pH 8, 500 mM NaCl) with concentrations of NsrP-ECD in the range from 1.3 to 1.7 nM was prepared in a Hellma Macro-cuvette 100-QS. 10–50 μL of 20 mM bacitracin stock solution in H_2_O was added to the cuvette. The cuvette was placed into the fluorolog sample holder and the reaction mixture was incubated for 2 min while stirring before measuring the fluorescence intensity. The measurements were conducted for bacitracin concentrations in the range from 0 to 2.8 mM. For measurements with Zn^2+,^ 100 mM of ZnCl_2_ was added to the 20 mM bacitracin stock solution.

In this experiment, the fluorescence of tyrosine was monitored because *Sa*NsrP ECD lacks tryptophan residues. 260 nm and 304 nm were used as excitation and emission wavelengths, respectively. The resulting fluorescence signal peak maximum was normalized and showed a decrease upon bacitracin binding. This difference was plotted as 1-F(%) against the bacitracin/Zn-bacitracin concentration. Finally, the K_D_ was determined with GraphPad Prism version 9.5.1 for MacOS, GraphPad Software, San Diego, California USA. Measurements were performed in triplicates.

### 2.6 Small angle X-ray scattering of *Sa*NsrP ECD and *Sa*NsrFP

We collected the data of *Sa*NsrP ECD and *Sa*NsrFP on the beamline BM29 at the ESRF Grenoble (Tully et al., 2023). The BM29 beamline was equipped with a PILATUS 3 × 2 M detector (Dectris) at a fixed distance of 2.813 m. The measurements were performed with a *Sa*NsrP ECD (without His-tag) concentration of 1.65 mg/mL and *Sa*NsrFP concentration of 0.5 mg/mL, respectively, at 10 °C with the corresponding buffers as background. We collected 10 frames with an exposer time of 1 second per frame and scaled the data to absolute intensity against water. We checked each frame for radiation damage using CorMap/χ^2^ test, implemented in PRIMUS (Konarev et al., 2003).

All used programs for data processing were part of the ATSAS Software package (Version 3.1.3) (Manalastas-Cantos et al., 2021). Primary data reduction was performed with the program PRIMUS (Konarev et al., 2003). With the Guinier approximation (Guinier, 1939), we determine the forward scattering *I(0)* and the radius of gyration (*R_g_*). The program GNOM (Svergun, 1992) was used to estimate the maximum particle dimension (*D_max_*) with the pair-distribution function *p(r)*. We calculated an ab initio model of *Sa*NsrP ECD with GASBOR (Svergun et al., 2001) and superimposed it with an AlphaFold3 (Abramson et al., 2024) model using SUPCOMB (Kozin and Svergun, 2001). All results are displayed in Supplementary Table 1.

### 2.7 Computational predictions of the bacitracin binding mode

A full model of the *Sa*NsrFP protein was generated with ColabFold (Mirdita et al., 2022) using 12 recycling cycles and creating four independent models. A sequence search for MSA construction was performed against Mgnify, UniRef, and PDB70 databases. The final model was chosen based on the resulting pLDDT score (Supplementary Figure 5). The extracellular domain comprising the residues between the amino acids N307 and L517 was taken for further experiments. The final model has been uploaded to https://www.modelarchive.org/doi/10.5452/ma-m0kcc.

Extensive conformational sampling of bacitracin was carried out using the MacroModel tool in Schrödinger (Watts et al., 2014) based on the protocol used in previous macrocycle sampling benchmarks (Alogheli et al., 2017). The initial structure of bacitracin was taken from the PDB entry 4K7T. Protonation states for side chains were assigned using Propka (Rostkowski et al., 2011). 1,000,000 steps of the Monte Carlo Multiple Minimum (MCMM) search were performed incorporating distance restraints between the peptide and the bound zinc ion. Extended sampling was allowed by incorporating the sampling of torsional angles of amides, esters, C-N and N-N single bonds, as well as C=N and N=N double bonds. A wide-opening ring criterion was used (0–100 Å) avoiding atoms adjacent to stereocenters. 50,000 steps of truncated Newton’s conjugate gradient (TNCG) method were performed for energy minimization. Redundancy within the resulting conformers was removed using an RMSD criterion of 0.5 Å for heavy atoms. This resulted in 67,949 unique conformations. The zinc ion was removed before moving on to docking experiments to prevent steric clashes with the receptor.

A putative binding region within the extracellular loop domain was identified using Schrodinger’s SiteMap (Halgren, 2009). A 46 Å Grid was manually placed within the site. Rigid docking was carried out using Glide in standard precision (Repasky et al., 2007) mode, including a reward for intramolecular hydrogen bonds. The resulting poses were filtered according to their docking energy score. Poses with energies below −6.0 kcal mol^−1^ were kept for further analyses. The final poses were clustered with cpptraj (Roe and Cheatham, 2013) using the DBScan algorithm with an RMSD-based cut-off of 2 Å for heavy atoms and three minimum points. Statistical assessments were performed using SciPy and NumPy in Python 3.10.

Parameters for the non-canonical residues of bacitracin were derived for the AMBER ff14SB force field using a fragmentation approach. Custom parameters were developed for D-ornithine (ORN), an isopeptide-linked lysine (LYX), and the N-terminal thiazoline-isoleucine moiety in its neutral (ICN) and protonated (ICP) states. For each residue, partial atomic charges were obtained via a multi-conformer Restrained Electrostatic Potential (RESP) (Wang et al., 2000) fitting protocol using capped model compounds. Low-energy conformers were generated with GOAT (De Souza, 2025) (ORCA 6.1), optimized in Gaussian 16 at the HF/6-31G(d) level of theory, and validated as true minima via frequency calculations. After excluding structures with intramolecular hydrogen bonds, Merz-Singh-Kollman electrostatic potentials were calculated [HF/6-31G(d)] and fitted using the *respgen* and *resp* programs in AmberTools22.

The specific models and constraints were tailored to each residue. For LYX, an *N*-acetyl-*N*_ε_-acetyl-l-lysine-*N*′-methylamide model was used to represent the isopeptide environment, with charges for five conformers fitted under the constraint that both capping groups and the total residue charge were zero. The fitting for ORN (*N*-acetyl-D-ornithine-*N*′-methylamide) used four conformers with a total charge constraint of +1. The ICN (charge 0) and ICP (charge +1) residues each used a single low-energy conformer for fitting. While most bonded parameters were assigned by analogy from ff14SB and GAFF2, a custom frcmod file was created to define specific parameters for the novel thiazoline ring structure. These terms were derived using the *parmcal* utility, transferred from the GAFF2 force field, or estimated based on chemical similarity. The final bacitracin molecule was assembled in tLEaP, where the isopeptide bond between the LYX^6^ side chain and the Asn^12^ C-terminus was manually defined.

All atom molecular dynamics simulations were performed using the Amber22 suite of molecular simulation software (Case et al., 2005). Topology files were generated with tleap (Case et al., 2023), using ff14SB as protein forcefield. The N and C terminus of the ECD were capped to reproduce the local environment of a non-truncated protein. The complexes were solvated in a TIP3P truncated octahedron box extending 12 Å from the protein. Cl^−^ counter ions were added to maintain electroneutrality of the simulation box. Three rounds of minimization were performed, first placing heavy positional restraints (5 kcal mol^−1^ Å^−2^) on all solute heavy atoms, then it was reduced to 0.1 kcal mol^−1^ Å^−2^, and then with no restraints. Each minimization round consisted in 500 steps of steepest descend, followed by 2000 steps of conjugate gradient. The systems were heated to 100 K in a timeframe of 25 ps in NVT conditions, using the Langevin thermostat with a collision coefficient of 1 ps^−1^. The systems were pressurized under NPT conditions using the Berendsen barostat with isotropic position and brought to a final temperature of 300 K over a time of 250 ps. All simulations were performed with a timestep of 2 fs, constraining bonds with hydrogen atoms using SHAKE (Kräutler et al., 2001) applying the GPU-accelerated implementation of the Particle Mesh Ewald Method (Salomon-Ferrer et al., 2013). Production runs of 0.5 μs were performed for the best scoring pose of each of the five best docking solution clusters. Post processing of trajectories was done using cpptraj. The RMSD of the macrocyclic region and the N-terminal region were calculated by fitting only the ECD with the *rms* command. We calculated the RMSD only for the center of mass of the atoms that make up the macrocycle backbone, and the backbone and thiazoline atoms that make up the linear N-terminus of bacitracin. Ligand occupation density was calculated using the volmap tool in VMD (Humphrey et al., 1996), calculating the average across all replicas. The macrocyclic region and N-terminal regions were defined using only their backbone heavy atoms. Representative poses of the C0 and C1 trajectories, respectively, were obtained by performing a hierarchical agglomerative clustering with an epsilon value of 2.5 Å in cpptraj.

### 2.8 Searching the AlphaFold protein structure database

The AlphaFold database (Jumper et al., 2021; Varadi et al., 2022; Varadi et al., 2024) was screened for BceAB-type ABC transporters. Parameters looked for were the characteristic domain architecture found for BceAB transporters of 10 transmembrane helices and a large extracellular domain (150–250 amino acids) located in between transmembrane helices 7 and 8 (Collins et al., 2010). Furthermore, the nucleotide-binding domain BceA should be located next to the gene encoding the transmembrane protein. BceB permease models were found under different names such as BceB, Bacitracin-export permease, FtsX-like permease, and FtsX domain-containing protein.

### 2.9 Structural alignments

Permeases of the different BceAB transporters from the AlphaFold database were cropped so that only the SABRE and Porter domain of the ECD with the first helical turn of the stalk helices remained. The structural alignment was performed by using the cealign tool of Pymol Version 2.5.4. The sequence alignments were performed by clustalO EMBL server (Madeira et al., 2024) and the sequence identity matrix was calculated with BioEdit V7.7.

## 3 Results

### 3.1 *In vitro* characterization of *Sa*NsrFP

**Figure 2:**
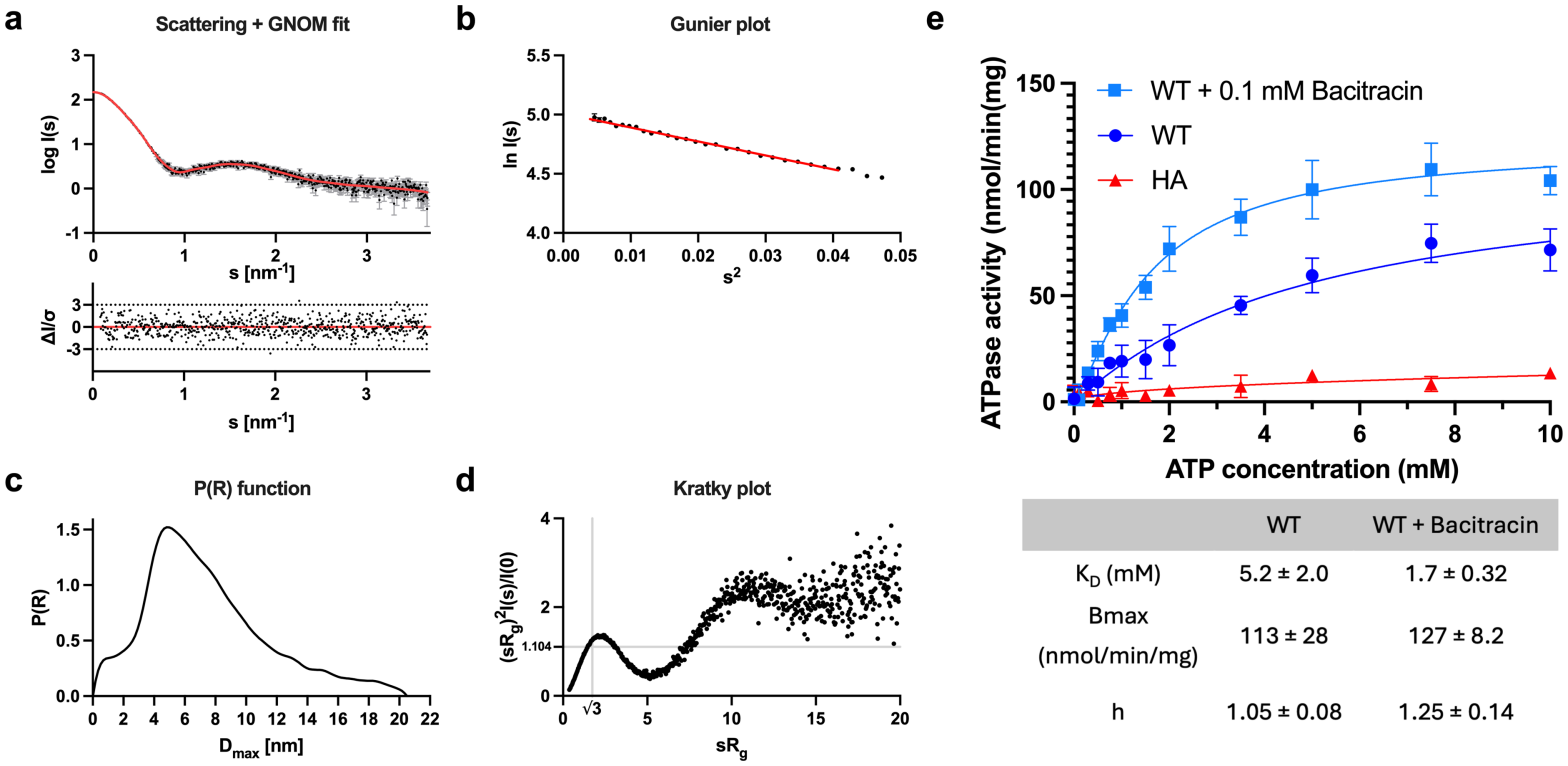
Characterization of *Sa*NsrFP. **(a)** Scattering data of *Sa*NsrFP. Experimental data are shown in black dots, with grey error bars. The GNOM fit is shown as red line and below is the residual plot of the data. **(b)** The Guinier plot of *Sa*NsrFP. **(c)** The *P(R)* function of *Sa*NsrFP indicates an elongated particle. **(d)** Dimensionless Kratky plots of *Sa*NsrFP. **(e)** Shown is the ATPase kinetic recorded for *Sa*NsrFP (WT) (dark blue), the ATP-deficient mutant *Sa*NsrF_H202A_P (red), and the WT with 100 μM bacitracin (light blue). All measurements were done at least in duplicates and background measurements without added magnesium were subtracted from all absorbance values. A final MgCl_2_ concentration of 10 μM and 2.1 μg SaNsrFP was mainted in all samples. Error bars denote standard deviation. Graphs were fitted using GraphPad Prism version 10.3.1 for MacOS, GraphPad Software, San Diego, California USA, www.graphpad.com.

To characterize the transporter *in vitro*, we expressed and purified *Sa*NsrFP from *E. coli*. After membrane isolation, membranes were solubilized with LMNG and subjected to an IMAC and SEC. Analyzing the elution fraction by SDS-PAGE analysis revealed pure bands above 55 kDa for *Sa*NsrP and 25 kDa for *Sa*NsrF (Supplemenatry Figure 1a). The results from SAXS (Figures 2a–d) show that *Sa*NsrFP is folded and contains no aggregates, as shown by the kratky plot (Figure 2d). The molecular weight calculated from the SAXS scattering is 148.30 kDa and suggests a fully assembled *Sa*NsrFP (135 kDa + detergent micelle) protein sample in a stoichiometry of 2:1 (Supplementary Table 1). ATPase assays revealed that *Sa*NsrFP displays a typical ATPase kinetic with a B_max_ of 113 ± 28 nmol/min/mg, a K_D_ of 5.2 ± 2.0 mM, and a Hill coefficient of 1.05 ± 0.08 (Figure 2e). Next, we set out to test whether bacitracin is capable of stimulating ATPase activity of *Sa*NsrFP. The addition of 100 μM bacitracin resulted in a B_max_ of 127 ± 8.2 nmol/min/mg, a K_D_ of 1.7 ± 0.32 mM, and a Hill coefficient of 1.25 ± 0.14 (Figure 2e). Highlighting, that the addition of bacitracin results in positive cooperative binding of ATP in the NBD, while it decreases the K_D_ by 3.1-fold. To confirm that the activity observed directly correlates to *Sa*NsrFP, we expressed, purified, and tested the ATPase-deficient mutant *Sa*NsrF_H202A_P (Furtmann et al., 2020), which showed only marginal ATPase activity (Figure 2e).

### 3.2 *In vitro* characterization of the ECD of *Sa*NsrP

To verify that the *Sa*NsrP-ECD is capable of binding bacitracin, we cloned and subsequently expressed the *Sa*NsrP-ECD without stalk helices (amino acids 307–517) in *E. coli*. Subsequently, purified *Sa*NsrP-ECD subjected to size exclusion chromatography (SEC) revealed a single homogenous peak (Supplementary Figure 1b). The purification was monitored via SDS-PAGE analysis (Supplementary Figure 1b).

**Figure 3:**
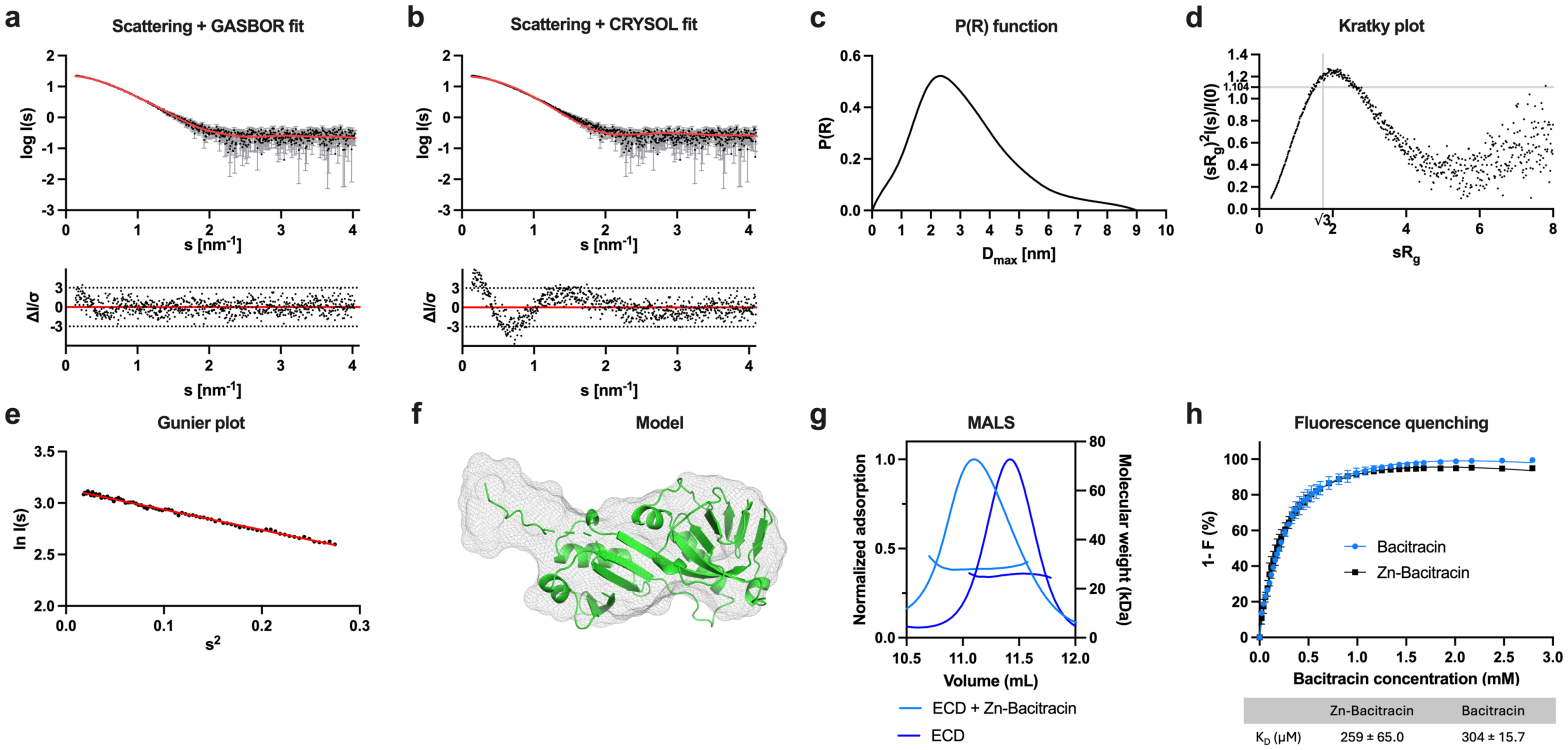
Characterization of the ECD of *Sa*NsrP. **(a)** SAXS scattering data for monomeric SaNsrP-ECD without His-tag. Experimental data are shown in black dots, with grey error bars. The GASBOR ab initio fit is show as a red line and below is the residual plot of the data. **(b)** Scattering data of *Sa*NsrP-ECD. Experimental data are shown in black dots, with grey error bars. The CRYSOL comparison fit from the AlphaFold3 model is shown as red line and below is the residual plot of the data. **(c)** The *P(R)* function of *Sa*NsrP-ECD indicates a globular particle with an extension. **(d)** Dimensionless Kratky plots of *Sa*NsrP-ECD. **(e)** The Guinier plot of *Sa*NsrP-ECD. **(f)** AlphaFold3 model of *Sa*NsrP-ECD superimposed with the GASBOR ab-initio model. The AlphaFold3 model is shown in green cartoon representation and the GASBOR ab-initio model is shown in grey mesh representation. **(g)** MALS measurement of *Sa*NsrP-ECD (blue) and *Sa*NsrP-ECD with the addition of Zn-Bacitracin (light blue). The normalized UV absorption at 280 nm and molecular mass were plotted against the elution volume. **(h)** Relative quenching of tyrosine fluorescence intensity of *Sa*NsrP-ECD with bacitracin (blue) and Zn-bacitracin (black). The measurements were conducted in the range of 304 nm and 0–2.79 mM bacitracin was added subsequently to the protein. Dissociation constants were calculated and graphs were fitted using GraphPad Prism version 9.5.1 for MacOS, GraphPad Software, San Diego, California USA, www.graphpad.com.

*Sa*NsrFP ECD showed a band lower than 25 kDa, which is in line with the theoretical mass of 23.8 kDa (Supplementary Figure 1b). Thus, we can show the stable and successful expression and purification of the ECD of a BceAB-type ABC transporter. To corroborate the molecular weight and oligomeric state of the protein sample, SAXS was performed, confirming that the ECD of *Sa*NsrP is a monomer in solution (Figures 3a–f; Supplementary Table 1 gives an overview of the results obtained by the SAXS measurement).

To confirm the interaction between bacitracin and the ECD of *S. agalactiae*, we used two independent methods: (1) Multi-Angle Light Scattering (MALS) and (2) intrinsic tyrosine fluorescence of the *Sa*NsrP-ECD (containing 9 tyrosines), which is altered upon bacitracin binding. The peak of the *Sa*NsrP-ECD without Zn-bacitracin (Figure 3g, blue) showed a molecular weight of 25.8 kDa in the MALS measurement, whereas the peak of *Sa*NsrP-ECD with Zn-bacitracin (1.5 kDa) (Figure 3g, light blue) shifted and contains particles of 28 kDa. Thus, the difference and the peak shift indicates binding of bacitracin (Figure 3g). Monitoring the tyrosine fluorescence of *Sa*NsrP-ECD upon the addition of bacitracin or Zn-bacitracin reveals a decrease in fluorescence with increasing concentrations (Figure 3h). For Zn-bacitracin, a K_D_ of 259 ± 65.0 μM was obtained and for bacitracin, a K_D_ of 304 ± 15.7 μM was obtained. This is significantly higher than the observed interaction of bacitracin with the full-length BceAB transporter from *B. subtilis* where bacitracin is bound with a K_D_ of 60 nM (Dintner et al., 2014). This difference might be due to the *in vitro* approach of our setup, where the transmembrane domain of *Sa*NsrP embedded in a lipid environment is lacking.

### 3.3 Molecular modeling prediction of bacitracin binding to *Sa*NsrP-ECD

In order to propose a bacitracin binding mode for *Sa*NsrP, we first used SiteMap to identify putative binding sites within it. This revealed two sites: one located within the transmembrane region near the extracellular side, and a second one placed in the central cleft of the ECD, between its two constituent subdomains, the SABRE and Porter subdomains (Supplementary Figure 2). Since the fluorescence quenching experiments showed that the ECD can bind on its own to bacitracin, all the key interactions between bacitracin and the protein must occur through the second site. Therefore, we performed molecular docking centered at the ECD cleft.

Given the large size and many rotatable bonds of bacitracin, conventional flexible docking is unviable. Thus, we first generated over 60,000 unique ligand conformations with MacroModel and then docked them into the *Sa*NsrP-ECD using rigid docking in Glide. The resulting poses were filtered according to their docking score (< −6 kcal mol^−1^) and clustered according to their RMSD. Only the five most populated clusters were considered for further analysis (Supplementary Figure 3).

In two of these clusters, the macrocyclic ring of bacitracin is placed within the central cleft of the ECD, while the thiazoline-containing N-terminus is placed at the edge, pointing towards the transmembrane domain (Supplementary Figure 6a, C0 and C3). In the three remaining clusters, the placement of bacitracin is reversed, with the N-terminus placed at the center of the ECD and the ring pointing toward the membrane surface (Supplementary Figure 6a, C1, C2 and C4). In all five clusters, bacitracin is in an extended conformation, with the N- and C-terminus spread apart from each other. These conformational states differ from the one in which bacitracin binds its lipidic target, where both ends come close together to surround the pyrophosphate moiety in the target, leading to a compact globular conformation (Supplementary Figure 4).

The most populated cluster (cluster C0), where bacitracin binds with its macrocyclic C-terminus placed at the central cleft of the protein, neither yields the best median binding energy nor the best scoring single pose. The best median binding energy is found in cluster C4 (−8.05 kcal·mol^−1^) and the single best scoring pose is in cluster C1 (−10.8 kcal·mol^−1^). In contrast to C0, C1 and C4 contain poses where the N-terminus is at the central cleft of the protein and the macrocycle is situated at the edge, suggesting that such poses are energetically more favorable. To further corroborate whether there are systematic binding energy differences between the two possible macrocycle orientations, we divided all the poses of the top five clusters into the two groups (Supplementary Figure 6b). The resulting distributions of binding energies differ significantly (*p* = 1.02·10^−23^, non-parametric paired Mann–Whitney U test), with poses where the ring is at the edge and the free tail is at the center being more favorable.

To further assess the robustness of the predicted poses, we performed molecular dynamics simulations taking as a starting point the best pose of each cluster. To evaluate the degree of structural variance throughout the simulations, we calculated the RMSD of the center of mass of the macrocyclic ring and the RMSD of the backbone atoms of the tail. The results show some conformational variability. Nonetheless, in poses starting with the macrocycle at the center (C0 and C3, Supplementary Figure 7), the macrocycle remained within the starting site in most of the replicas (9 out of 10 and 8 out of 10, respectively), while the N-terminus fluctuates widely (Supplementary Figure 8). The opposite trend was observed for poses with the N-terminus at the cleft, (C1, C2 and C4), albeit with an even larger fluctuation margin. Among the assessed poses, the least structural variance for the poses with the macrocycle at the center was observed starting from the binding pose C0, and for the poses with N-terminus at the center, it was observed for C1.

**Figure 4:**
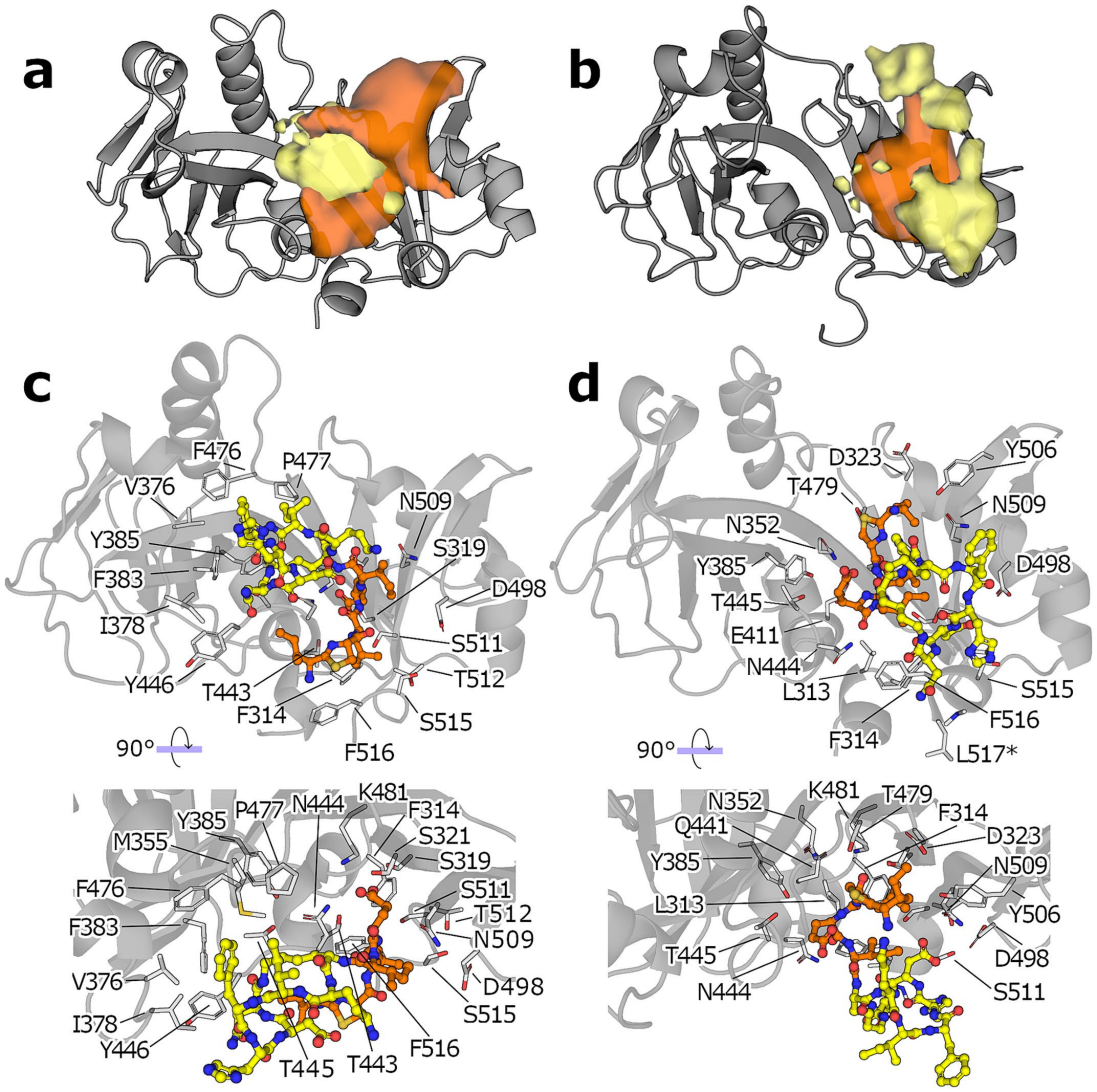
MD simulations of the ECD: bacitracin complex. **(a)** Occupancy of the macrocyclic region (yellow blob) and the N-terminus (orange blob) of bacitracin throughout the MD simulations starting from the pose C0. **(b)** Same as in **(a)** but for simulations starting from pose C1 (protein shown a gray cartoon). All isosurfaces are shown at the same sigma level (0.07). **(c)** Binding mode of the most populated cluster derived from the MD ensemble started from C0. **(d)** The protein is shown as gray cartoon, the amino acid side chains closer than 4 Å to any bacitracin atom are shown as white sticks and numbered according to the full length NsrP protein, the macrocyclic region of bacitracin is shown as yellow.

To analyze where on the protein surface each segment of bacitracin spent most of the simulation time, we performed a density analysis over both simulations sets. This is in essence a 3D histogram that shows the most occupied regions during an aggregated simulation time of 5 μs. For the simulations starting from C0, the macrocycle spends most of the time at the central cleft, close to where it was initially placed by molecular docking (yellow blob, Figure 4a), whereas the tail occupies two regions preferentially, one tucked under the ring near the SABRE subdomain, and the other one extended over the Porter subdomain (orange blob, Figure 4a). Similarly, the simulation of C1 shows the N-terminus occupying preferentially a wide region within the central cleft, slightly closer to the Porter subdomain (yellow blob, Figure 4b). However, the macrocyclic ring shows a less defined preferential region, with sparse densities observed above and below the position of the N-terminus. Finally, we derived a representative pose for the conformational ensembles sampled during the MD simulations by clustering according to the RMSD of the bacitracin backbone atoms; the resulting poses for the simulations started from C0 and C1 are shown in Figures 4c,d, respectively. Compared to the starting poses, bacitracin adopts a more compact conformation after MD simulation. For C0, the macrocyclic ring sits in a dominantly hydrophobic region of the SABRE domain, while the N-terminus points downwards, where the membrane would be located. On the other hand, C1 shows the N-terminus placed in a region close to the Porter subdomain rich in polar amino acids, while the ring remains detached from the protein, exposed to the solvent.

### 3.4 Structural identification of other BceAB-type transporters

**Figure 5:**
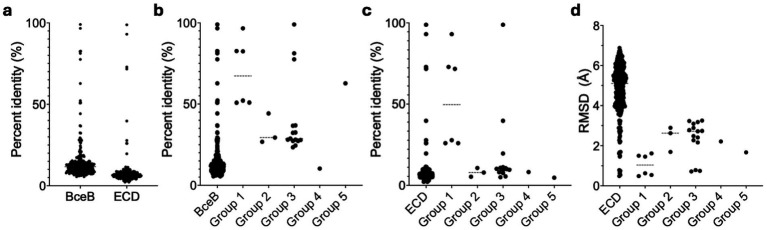
Statistical evaluation of sequence and structure conservation among BceBs and ECDs. **(a)** Represented is the sequence identity of the mutual alignment of all 24 BceBs/ECDs. The group-specific alignments for the whole BceB and the ECD are highlighted in **(b,c)** respectively. **(d)** The mutual RMSD values obtained with the cealign tool of Pymol are displayed for all ECDs and the respective groups. Dashed lines represent averages. Graphs were fitted using GraphPad Prism version 10.3.1 for MacOS, GraphPad Software, San Diego, California USA, www.graphpad.com. *n* = 276 (all BceBs/ECDs), 6 (Group 1), 3 (Group 2), 15 (Group 3) and 1 (Group 4 and 5).

We then investigated whether our results could be extended to other members of the BceAB transporter family. BceAB-type transporters are mainly found in Firmicutes bacteria (Dintner et al., 2011; Kobras et al., 2020). Since sequence conservation is low (Figure 5a), standard BLAST searches based on the *Sa*NsrFP sequence did not yield any hits. Hence, we used the AlphaFold Protein Structure Database (Jumper et al., 2021; Varadi et al., 2022) to search for other BceAB-type transporters applying structural alignments. We identified several BceAB-type ABC transporters in the genomes of different organisms including opportunistic pathogens as well as medically relevant human pathogens and *ESKAPE* organisms listed by the WHO (Tacconelli et al., 2018), such as *S. pneumonia*, *MRSA*, and *E. faecium* (Supplementary Table 2). A comparison of the predicted structures showed that the transmembrane domain of all BceB is structurally conserved. In contrast, many different structures are found for the ECDs (Supplementary Figure 9).

### 3.5 Structural alignments of ECDs reveal five distinct groups

The sequence identity observed for the ECD is lower than that of the whole BceB (Figure 5a). Therefore, we constructed structure-based alignments of only the ECD by removing the TMDs of the different BceAB-type transporters found in the AlphaFold database, leaving only the SABRE and Porter subdomains of the ECD with the first helical turn of the stalk helices. ECDs with approximately 180–230 amino acids were used for structural alignments. We determined RMSD values of the ECDs over 110–200 C*_α_* atoms with the cealign tool of PyMol.

**Figure 6:**
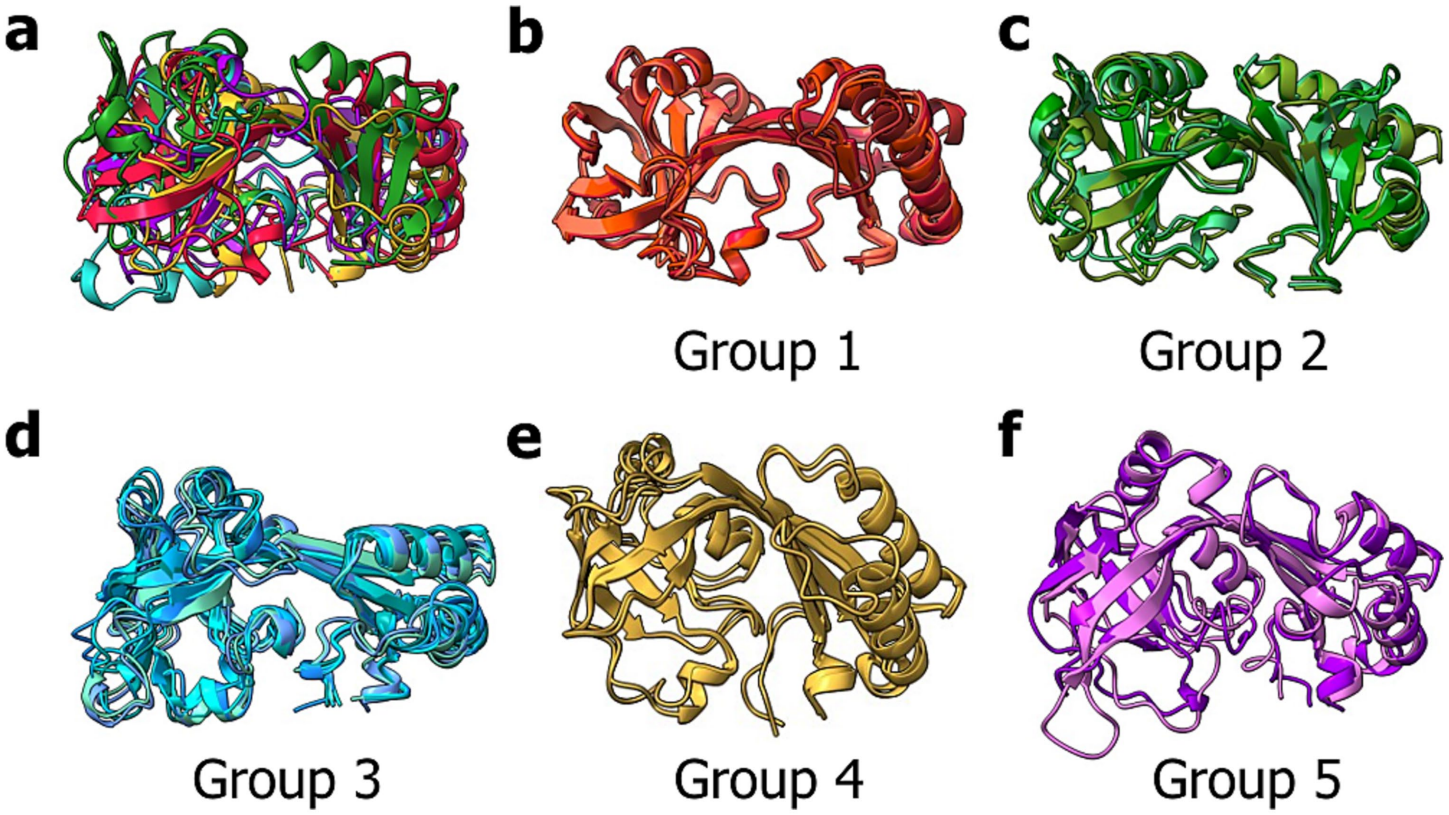
Superimposition of all ECDs. **(a)** Shown is the structural alignment of ECDs from BceB (*S. pneumoniae*, Group 1, red), BceB (*S. suis*, Group 2, green), BceB (*B. subtilis*, Group 3, blue), YvcS (*B. cereus*, Group 4, violet), YxdM (*B. subtilis*, Group 5, gold). Additional structural alignments are depicted for Group 1 **(b)**, Group 2 **(c)**, Group 3 **(d)**, Group 4 **(e)** and Group 5 **(f)**. Image was created using ChimeraX Version 1.8.

From all 24 ECDs, we were able to classify 17 ECDs into five groups (Supplementary Figures 9, 10). Based on this grouping, the sequence identity on the whole BceB level increases around 3.5-fold from below 20% to around 70% for group 1 and 5, while groups 2 and 3 show an 2-fold increase to a sequence identity below 40% (Figure 5b). For group 4 a sequence identity of below 20% is maintained. In contrast, the sequence identity of the ECD level is not affected and stays below 20% for all groups except group 1, which shows elevated sequence identity values around 50% (Figure 5c; Supplementary Figure 10). This however, does not correlate to the structural conservation, as the average RMSD within each group is 2-fold lower than the average of all RMSDs from all ECD to ECD comparisons. A average RMSD < 2.5 Å is maintained in all groups. This also correlates visually to the grouping of the AlphaFold models depicted in Figure 6. Group 1 is formed by four, group 2 by three, group 3 by six and group 4 and 5 by two ECDs each.

**Figure 7:**
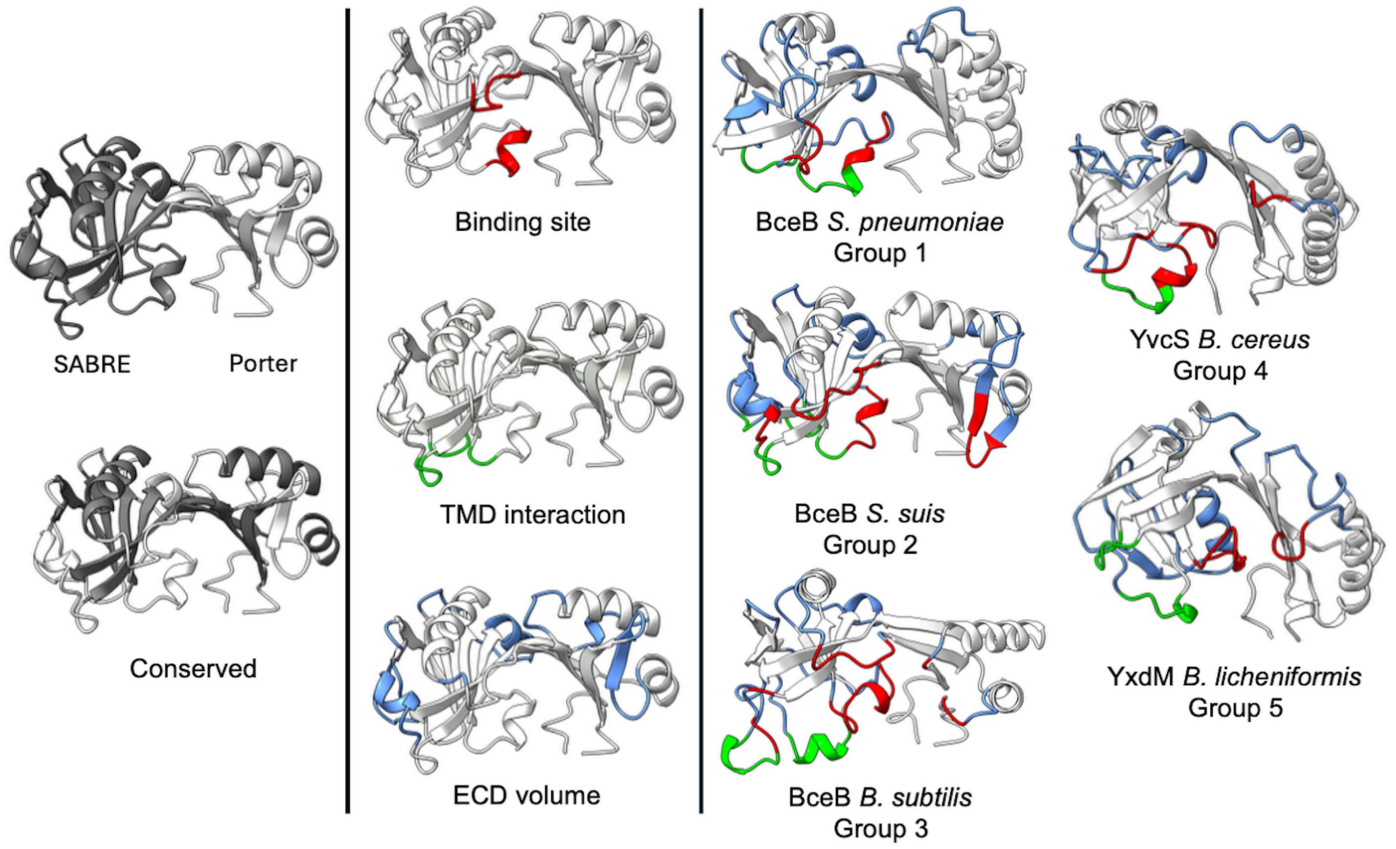
Structural classification of BceAB-type permeases. Depiction of the AlphaFold models of *Sa*NsrP and one representative for each of the five groups. The models show the conserved secondary structure elements (grey/white), as well as elements of category one (red), two (green), and three (blue). Image was created using ChimeraX Version 1.8.

Overall, conserved secondary structures are observed among all ECDs. In all ECDs, two *β*-strands connect the SABRE and Porter domains. The Porter subdomain is formed by two conserved β-sheets and two α-helices, while the SABRE subdomain contains three β-strands, a β-β-loop, and one conserved α-helix in front of one of the two domain-connecting β-strands (Figure 7, left). This core structure can then be elongated by a short stretch of α-helices and β-strand segments, which seem to be specific insertions for each group. These group-specific secondary structure elements can be classified into one of three categories.

The first category (Figure 7, red) includes α-helices and unstructured loops, which are directly involved in forming the binding interface of the ECD located below the domain-connecting β-strands, between the SABRE and Porter subdomain, where the ligand binding site is predicted to be. Typically, these elements originate from the conserved three β-strands in the SABRE subdomain and reach into the binding interface.

The second category (Figure 7, green) contains α-helices and unstructured loops originating below the conserved β-strands of the SABRE subdomain. These structures are most likely directly involved in interactions with TMH 5–6, helix 9–10, and the binding interface of the ECD and might play a role in signal transduction of a binding event. Thus, the size and position might be directly related to TMH 5–6 and helix 9–10 architecture.

α-helices and β-strands of the third category (Figure 7, blue) expand the overall shape of the ECD but do not influence the binding site and do not contribute to a possible interaction with the TMD.

As the size and position of these elements are group-specific, they might explain how substrates are recognized and how substrate binding can differently affect the full-size transporter.

This highlights that the structure of the ECD might provide a scaffold that restricts the volume of the binding interface of the ECD and, therefore, the bulkiness of substrates recognized, while the specific sequence is responsible for substrate interactions and the overall charge of the binding interface.

## 4 Discussion

In recent work, *L. lactis* NZ9000 cells expressing *Sa*NsrFP showed the highest resistance against bacitracin while conferring lower resistance against cationic antimicrobial peptides such as nisin in comparison to the sensitive empty vector control strain (Gottstein et al., 2022). It was concluded in the same study that there is a first-line resistance mechanism against bacitracin by directly binding it or shielding the target UPP. This gives the cell time to react and to rebuild the peptidoglycan layer which becomes impermeable for several structurally diverse cationic antimicrobial peptides. This appears to be a second line of defense against antimicrobial peptides and is coupled to the binding of bacitracin to *Sa*NsrFP and subsequent ATP hydrolysis since the ATP-deficient variant does not show this phenotype.

To gain further insight into how *Sa*NsrFP operates, we expressed and purified the BceAB-type ABC-transporter in LMNG micelles and investigated its ATPase activity. The results show that the WT transporter is active and has a maximal ATPase activity of 135 ± 32.7 nmol/min/mg, while the mutant *Sa*NsrF_H202A_P is showing only marginal activity. This is in line with the *in vivo* data for *Sa*NsrFP, where the ATPase-deficient mutant lacks resistance (Reiners et al., 2017), as well as the *in vitro* characterization of *Sa*NsrF (Furtmann et al., 2020). Next, we probed the influence of bacitracin binding on the ATPase activity. Here, our results show that bacitracin stimulates *Sa*NsrFP ATPase by inducing cooperativity for ATP in the NDB and decreasing the K_D_ by 2.8-fold, while the B_max_ remains the same. This stimulation is in line with the work of Diagne et al. (2022), which shows ATPase stimulation of BceAB from *S. pneumonia* by bacitracin. In this study, the addition of bacitracin increased ATPase levels, but no comment was made regarding K_D_ or possible cooperativity. In contrast, BceAB from *B. subtilis* (George et al., 2022) showed ATPase inhibition by bacitracin. This suggests that there does not seem to be a uniform response to bacitracin in terms of ATPase activity.

The study of Cho et al. (2021) showed that by swapping the ECD the substrate specificity is altered, which suggests that the ECD is involved in substrate recognition. To confirm this, we successfully expressed and purified the *Sa*NsrP-ECD and demonstrated via SEC-MALS and via measuring intrinsic tyrosine fluorescence that it binds bacitracin. The observed K_D_ shows minimal variation in the presence or absence of zinc ions, implying that the interaction is not influenced by bacitracin conformations.

Putative binding site prediction by SiteMap revealed two distinctive sites at each region of *Sa*NsrP: One at the transmembrane region and another one at the ECD. The presence of two binding sites in NsrP is consistent with the mechanistic model proposed for the homologous protein BceAB (George et al., 2022), which postulates that this transporter detaches the lantibiotic from its lipidic target by first sequestering the complex through interactions with the transmembrane domain, followed by a direct interaction with the lantibiotic occurring through the ECD.

The docking results revealed two main possible binding orientations for bacitracin: Either the macrocyclic ring or the N-terminus of bacitracin is at the center of the ECD (between the Porter and SABRE domains) of which the latter orientation has significantly better scores. Performing MD simulations of the complexes showed some structural variation for bacitracin. Poses in which the macrocycle was at the central cleft showed a more firmly bound macrocyclic region and a highly variable N-terminus region and vice versa. Overall, non-tight binding of bacitracin to the ECD with high conformational fluctuations is not surprising given the binding affinity observed for the complex in the micromolar range. That we observe two putative binding modes for the ligand could be either related to the fact that *Sa*NsrP is not a selective protein and confers resistance against structurally different peptides such as nisin and bacitracin, indicating that it can bind different peptidic moieties. Alternatively, it is also possible that the ECD binds to bacitracin in different ways through the catalytic cycle required to detach it and release it from LIPID II. It is also worth noting, that the significant difference between the K_D_ values observed during our characterization of the ECD compared to the previously reported values for the whole complex (Dintner et al., 2014) suggests that there may be an allosteric contribution of the transmembrane regions to the conformational landscape of the ECD, indicating that the conformation of the ECD may significantly change during the entire catalytic cycle. Nonetheless, the results provide a forward step into constructing an atomistic model of the mechanism of action of *Sa*NsrFP.

Bacitracin resistance has been attributed to several other BceAB-type transporters in *B. subtilis*, *L. monocytogenes*, *S. mutans*, *S. aureus* (Gebhard, 2012), and *S. pneumoniae* (Diagne et al., 2022). We further showed that BceAB transporters can be found in opportunistic pathogenic and clinically pathogenic bacterial strains when using structural searches rather than sequence alignments. The presence of these transporters might be the cause of resistance against bacitracin and or other antimicrobial peptides.

Also, we could classify ECDs from BceB models from different bacteria into five groups, identifying conserved secondary structures in all (a set of two *β*-strands over a set of three β-strands in the SABRE domain, two conserved β-strands and *α*-helices in the porter domain, as well as two domain connecting β-strands) and individual secondary structure elements unique for each group. Comparing the latter ones between all ECDs enabled us to classify which secondary structures are responsible for generating the binding interface, forming interactions with the TMD, or modulating the ECD shape.

As resistance data for all categorized BceABs is limited (8 out of 17 are partially characterized), a direct correlation between the grouping and mediated resistance is not possible. However, the available data shows that overall BceABs respond to LL-37 (8 of 17 positive) and bacitracin (7 of 17 positive, VraFG negative) (Table 1).

**Table 1:** Resistance profile of all categorized BceAB-type ABC-transporters.

Group	Name & organism	Bacitracin	Nisin	LL-37	Vancomycin
1	BceB *S. pneumoniae*	Yes (Majchrzykiewicz A. et al., 2010)	N.a.	Yes (Majchrzykiewicz A. et al., 2010)	No (Diagne et al., 2022)
2	MbrB *S.mutans*	Yes (Tian et al., 2018)	No (Tsuda et al., 2002)	Yes (Tian et al., 2018)	N.a.
	NsrP *S. agalactiae*	Yes (Gottstein et al., 2022)	Yes (Gottstein et al., 2022)	Yes (Yang et al., 2019)	Yes (Gottstein et al., 2022)
3	BceB *B. subtilis*	Yes (Staron et al., 2011)	No (Staron et al., 2011)	Yes (Pietiäinen et al., 2005)	No (Staron et al., 2011)
	VraG *S. aureus*	No (Vestergaard et al., 2017)	Yes (Vestergaard et al., 2017)	Yes (Vestergaard et al., 2017)	Yes (Meehl et al., 2007)
	VraE *S. aureus*	Yes (Pietiäinen et al., 2005)	Yes (Popella et al., 2016)	Yes (Mensa et al., 2014)	No (Popella et al., 2016)
4	PdsB *B. subtilis*	Yes (Staron et al., 2011)	Yes (Staron et al., 2011)	Yes (Pietiäinen et al., 2005)	No (Staron et al., 2011)
5	ApeB *B. subtilis*	No (Staron et al., 2011)	No (Staron et al., 2011)	Yes (Pietiäinen et al., 2005)	No (Staron et al., 2011)

N.a., not applicable.

In our grouping both VraFG and VraDE are placed in Group 3. This is interesting, as Cho et al. (2021) demonstrated that the different resistance profiles regarding bacitracin are inverted if the ECDs of both transporters are swapped. Together with the low sequence identity of around 10%, this suggests that the shape of the ECD might guide the width of the substrate spectrum by restricting the volume in the ECD, while the specific sequence is required for fine-tuning the substrate spectrum. To advance this classification based on structure and sequence identity, more functional resistance data, *in vitro* binding assays, and especially more substrate-bound structures of BceABs are needed.

Conclusively, BceAB-type transporters such as *Sa*NsrFP are conserved in clinically relevant human pathogenic strains and nonpathogenic strains. Although not conserved at the sequence level, the topology of the protein and its cognate encoding operons are conserved. The results of this study are consistent with previous observations that *Sa*NsrFP confers resistance against bacitracin, and we were able to show that the ECD is the binds bacitracin.

## Data Availability

The datasets presented in this study can be found in online repositories. The names of the repository/repositories and accession number(s) can be found at: https://www.modelarchive.org/doi/10.5452/ma-m0kcc, https://www.sasbdb.org/data/SASDWZ6/ and https://www.sasbdb.org/data/SASDW27/.
