# Supplementary material for: The extracellular domain of SaNSrFP binds bacitracin and allows the identification of new members of the BceAB transporter family
Source: Front Microbiol. 2025 Sep 17;16:1662803. doi: 10.3389/fmicb.2025.1662803 (PMC12484072; doi:10.3389/fmicb.2025.1662803)
Supplement: Supplementary file 1 [file Data_Sheet_1.pdf]

Supplementary Information

The extracellular domain of *Sa*NSrFP binds bacitracin and allows the identification of new members of the BceAB transporter family

Christian Mammen[1], Julia Gottstein[1], Pablo A. Cea [2], Kira Tantsur[1], Jens Reiners[3], Michele Bonus[2], Holger Gohlke[2,4], Sander H. J. Smits[1,3*]

[1]Institute of Biochemistry, Heinrich Heine University Düsseldorf, Düsseldorf, Germany.

[2]Institute for Pharmaceutical and Medicinal Chemistry, Heinrich-Heine-University Düsseldorf, Düsseldorf, Germany.

[3]Center for Structural Studies, Heinrich Heine University Düsseldorf, Düsseldorf, Germany.

[4]Institute of Bio- and Geosciences (IBG-4: Bioinformatics), Forschungszentrum Jülich, Jülich, Germany

[*] **Correspondence:**
Sander Smits
Institute of Biochemistry
Heinrich Heine University Düsseldorf
Universitätsstr. 1
40225 Düsseldorf, Germany
Phone: +49(0)211-81-12647
Fax: +49(0)211-81-15310
Email: sander.smits@hhu.de

Keywords: BceAB, Antimicrobial resistance, Lantibiotic, ABC transporter, Human pathogen

Heterologous expression and purification of *Sa*NsrFP in *E. coli* (DE3) C41 dd

SaNsrFP (Uniprot ID: X5KGL2 + Q8DZX0) was cloned from pIL-sv-NsrFP into pET16b for heterologous expression of *Sa*NsrFP with an N-terminal 10x-His-tag at *Sa*NsrF. Next, chemically competent *E. coli* (DE3) C41 dd cells (Kanonenberg et al., 2019) were transformed with the plasmid pET16b-NsrFP. Transformed cells were grown in LB-medium at 37 °C and 180 rpm. The main cultures were inoculated to an OD_{600} of 0.1 and grown till OD_{600}= 0.6 at 37 °C and 180 rpm in baffled flasks. Protein expression was induced by the addition of 0.5 mM IPTG and cells and subsequently, the temperature was lowered to 18 °C. After overnight expression, cells were harvested by centrifugation at 5,000 xg, and cells were resuspended in resuspension buffer (300 mM NaCl, 50 mM Tris pH 8, 10 % Glycerol) supplemented with 1,000 U DNAse and one protease inhibitor tablet (Roche). Next, cells were lysed at 1.5 kbar with a cell disruptor (Microfluidics) and membranes were collected after two centrifugation steps at 20,000 xg and 150,000 xg respectively. Membranes were homogenized with resuspension buffer and stored at -80 °C. For purification, membranes were diluted 10-fold with purification buffer (100 mM NaCl, 50 mM HEPES pH 8, 10 % Glycerol) containing 1 % (w/V) LMNG. After 3 h of solubilization, non-solubilized material was removed by centrifugation at 20,000 xg. Successfully solubilized proteins were spiked with 10 mM imidazole pH 8 and loaded onto an equilibrated 1 mL Hi-trap IMAC column (Cytiva). First, a wash step was conducted with 100 mM imidazole followed by the elution of *Sa*NsrFP with 300 mM imidazole. Fractions containing *Sa*NsrFP were collected, concentrated with Vivaspin turbo 20 concentrators (100 kDA cutoff) and centrifuged at 100,000 xg prior to injection onto an equilibrated Superrose 6 Increase 10/300 GL column (Cytiva). During all purification steps, an LMNG concentration of 0.005 % was maintained.

Heterologous expression and purification of *Sa*NsrP-ECD in *E. coli* (DE3) BL21

*Sa*NsrP-ECD (aminoacids 311 to 512) was cloned from pIl-sv-NsrFP vector into pET28b with an N-terminal 6xHis tag. *Sa*NsrP-ECD was expressed in *E. coli* (DE3) BL21 in LB supplemented with 30 µg/mL kanamycin overnight at 18 °C. In short, 2 LB supplemented with 30 µg/mL kanamycin in baffled flasks were inoculated from an overnight culture to OD_{600} = 0.05 and incubated till OD_{600} = 0.3 at 37 °C and 180 rpm. Next, cultures were transferred to 18 °C and protein expression was induced by the addition of 1 mM IPTG once cultures reached OD_{600} = 0.8. After overnight expression, cells were harvested at 5,000 xg for 15 min at 4°C and resuspended in resuspension buffer (50 mM Tris pH 8.0, 50 mM NaCl, 10 % Glycerol) supplemented with 1000 U of DNAse, RNAse and one protease inhibitor tablet (Roche). After cell disruption at 1.5 kbar, cell debris and membranes were removed by ultracentrifugation at 150,000 xg for 1 h at 4 °C. The supernatant was spiked with 20 mM imidazole and loaded onto a HiTrap Chelating HP 5 mL column (Cytiva) loaded with Ni^{2+}. After loading, the column was washed with low IMAC buffer (20 mM Tris pH 8, 400 mM NaCl, and 20 mM imidazole) and 25 % high IMAC Buffer (20 mM Tris pH 8, 400 mM NaCl, 400 mM imidazole). Next, *Sa*NsrP-ECD was eluted with 100 % high IMAC Buffer. Next, a thrombin cleavage (Sigma) was performed according to the manufacturer's instructions. After reverse IMAC, *Sa*NsrP-ECD was concentrated with a 10 kDa cutoff concentration unit and centrifuged at 100,000 xg for 15 min. For SEC, *Sa*NsrP-ECD was injected onto a Superdex 75 10/300 column preequilibrated with SEC buffer (25 mM MES pH 6.0, 500 mM NaCl).

Supplementary Table 1: SAXS data for *Sa*NsrFP in LMNG micelles and *Sa*NsrP-ECD.

**Data collection parameters**		
SAXS Device	BM29, ESRF Grenoble (Tully et al., 2023)	
Detector	PILATUS 3 X 2M	
Detector distance (m)	2.813	
Beam size	200 µm x 100 µm	
Wavelength (nm)	0.099	
Sample environment	Quartz glass capillary, 1 mm ø	
Absolute scaling method	Comparison with scattering from pure H_2O	
Normalization	To transmitted intensity by beam-stop counter	
Scattering intensity scale	Absolute scale, cm^{-1}	
s range (nm^{-1})‡	0.06 – 5.3	
**Sample**	***Sa*NsrP ECD**	***Sa*NsrFP**
Organism	*S. agalactiae*	
UniProt ID	Q8DZX0	X5KGL2 + Q8DZX0
Mode of measurement	batch	
Temperature (°C)	10	
Exposure time (# frames)	1 (10 Frames)	
Protein buffer	25 mM MES pH 6.0, 500 mM NaCl	50 mM Tris pH 8, 300 mM NaCl, 10 % Glycerol, 0.005 % LMNG
Protein concentration (mg/ml)	1.65	0.5
**Structural parameters**		
Guinier Analysis (PRIMUS)		
$I(0) \pm \sigma$ (cm^{-1})	22.94 ± 0.06	148.30 ± 0.59
$R_g \pm \sigma$ (nm)	2.43 ± 0.01	5.83 ± 0.03
s-range (nm^{-1})	0.134 – 0.524	0.083 – 0.223
min < sRg < max limit	0.325 – 1.274	0.487 – 1.298
Data point range	1 - 77	1 - 28
Linear fit assessment (R^2)	0.994	0.995
PDDF/P(r) Analysis (GNOM)		
$I(0) \pm \sigma$ (cm^{-1})	23.01 ± 0.06	148.30 ± 0.54
$R_g \pm \sigma$ (nm)	2.50 ± 0.01	6.00 ± 0.03
D_{max} (nm)	9.00	20.50
Porod volume (nm^3)	50.17	734.55
s-range (nm^{-1})	0.134 – 4.187	0.083 – 3.664
$\chi 2$ / CorMap P-value	1.042 / 0.537	1.143 / 0.080
**Molecular mass (kDa)**		
From *I*(0)	22.94	148.30
From Qp (Porod, 1951)	24.92	268.04
From sequence	23.38	135.77
**Atomistic modeling**		
CRYSOL (with default parameters)		
Constant subtraction allowed		
Structure template	AlphaFold3 model	
s-range for fit (nm^{-1})	0.134 – 4.187	
χ^2, CorMap *P*-value	3.159 / 5.06e-34	
**Ab initio modeling**		
GASBOR		
Symmetry	P1	
s-range for fit (nm^{-1})	0.134 – 4.028	
χ^2, CorMap *P*-value	1.093 / 0.0057	

**SASBDB accession codes** (Kikhney et al., 2020)	SASDWZ6	SASDW27
**Software**		
ATSAS Software Version (Manalastas-Cantos et al., 2021)	3.0.5	
Primary data reduction	PRIMUS (Konarev et al., 2003)	
Data processing	GNOM (Svergun, 1992)	
Ab-initio modelling	GASBOR (Svergun et al., 2001)	
Model visualization	PyMOL (PyMOL, 2022)	

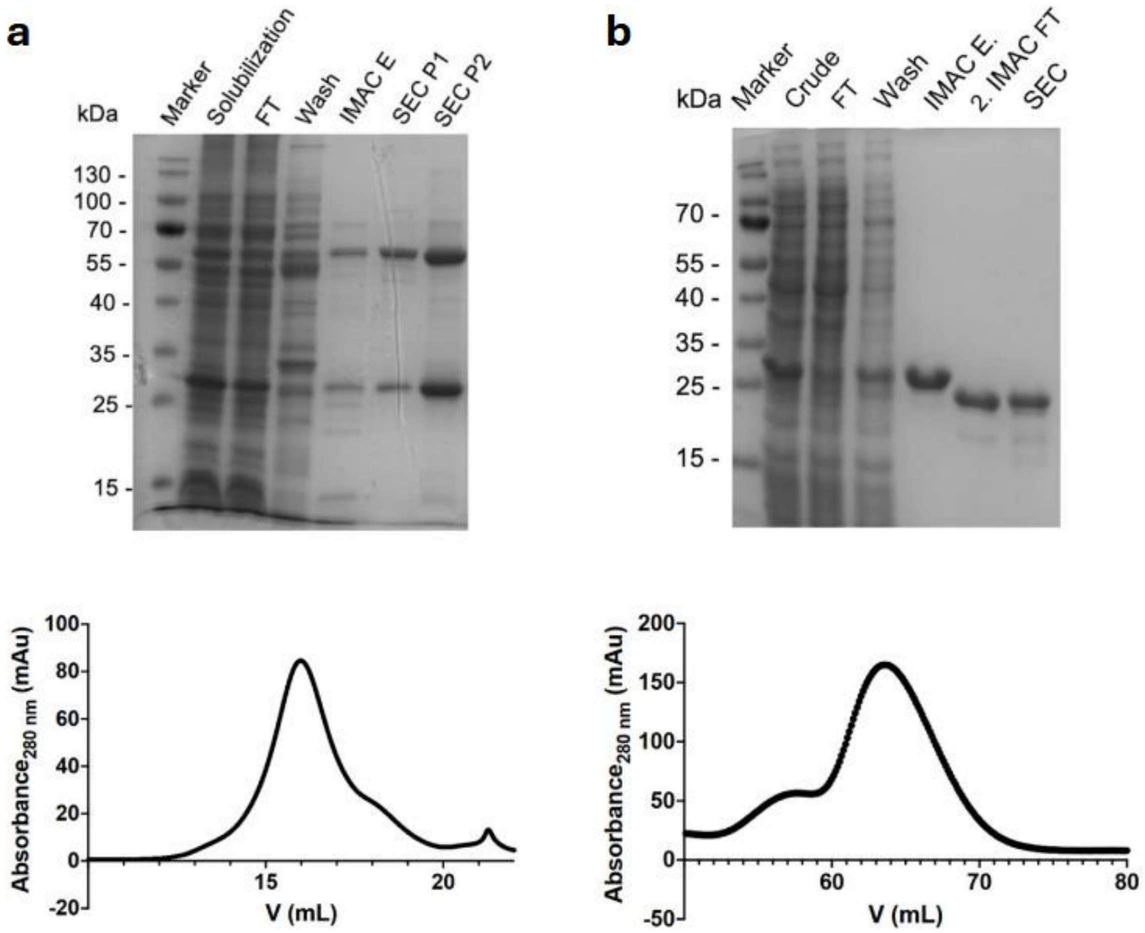

Supplementary Figure 1: SDS-PAGE and Size exclusion chromatogramsof *Sa*NsrFP (a) and *Sa*NsrP-ECD (b). a) 15% SDS gel showing the different fraction samples from the purification of *Sa*NsrP. Lane 1 shows the standard protein marker. Lanes 2-4 represent samples taken from the supernatant after solubilization with LMNG, flowthrough, and wash fraction. Lane 5 shows the IMAC eluate at 300 mM of imidazole, Lane 6 the SEC elution, which was used for all ATPase assays. **b)**15% SDS gel showing the different fraction samples from the purification of the short construct of the ECDsh of *Sa*NsrP. Lane 1 shows the standard protein marker. Lanes 2-4 represent samples taken from crude extract, flowthrough, and wash fraction. Lane 5 shows the IMAC eluate at 300 mM of imidazole, Lane 6 the flowthrough sample of the second IMAC after thrombin digestion, and Lane 7 the eluate sample of the SEC.

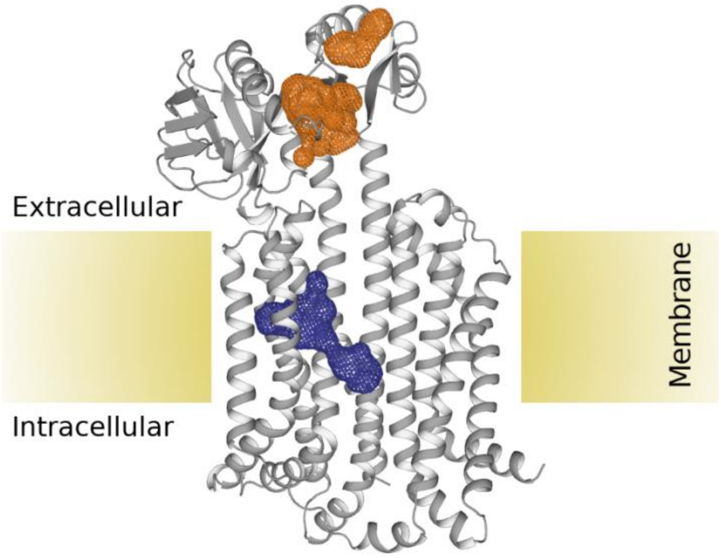

Supplementary Figure 2: Putative ligand binding sites on NsrFP. The putative binding site located in the transmembrane domain is shown as a blue mesh, whereas the putative binding site on the extracellular domain is shown as an orange mesh.

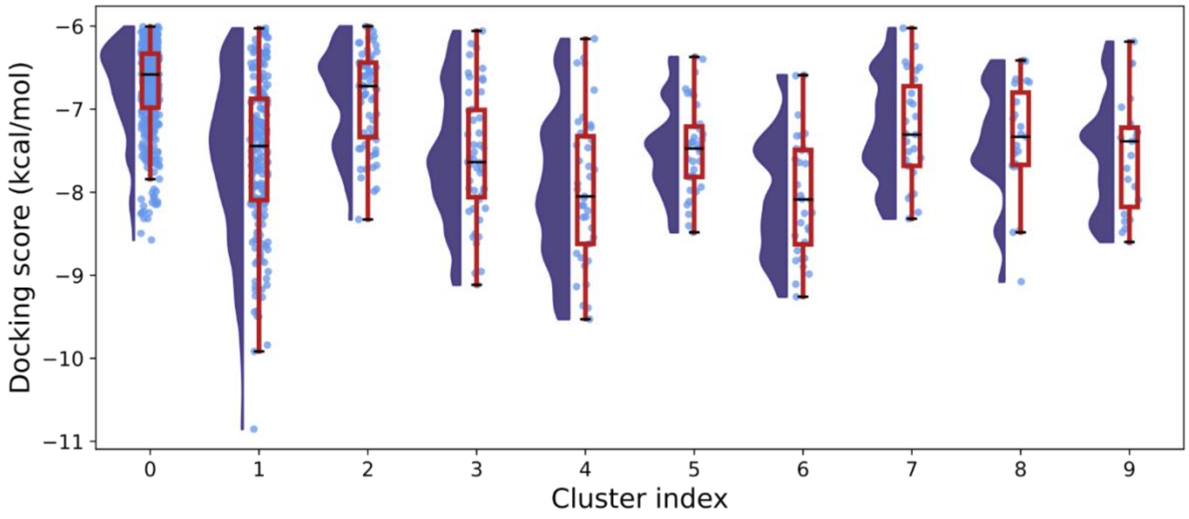

Supplementary Figure 3: Rain cloud plots showing the energy score distribution per cluster. The half-violin plots show the data density distribution (dark blue), each data point is shown in light blue. A box plot is overlayed on top (red). Its center line indicates the median of the distribution, the edges of the box show the lower and upper quartile and the whiskers show the minimum and maximum values of the distribution, excluding outliers (defined as points 1.5 times outside the difference between the lower and upper quartile).

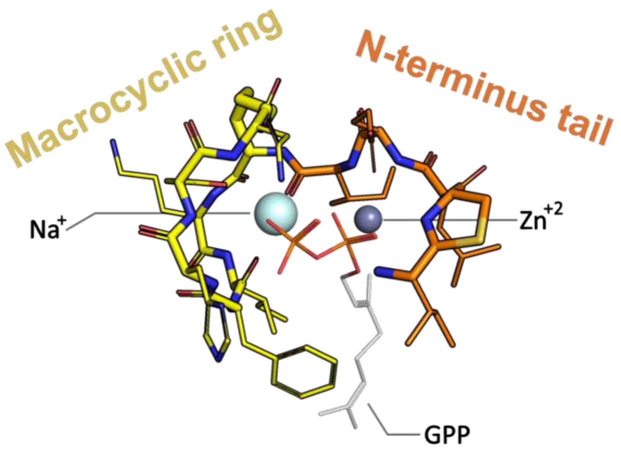

Supplementary Figure 4: Bacitracin bound to lipid II mimetic (GPP) (PDB 4K7T). The macrocyclic ring and the N-terminus of the peptide are colored yellow and orange, respectively.

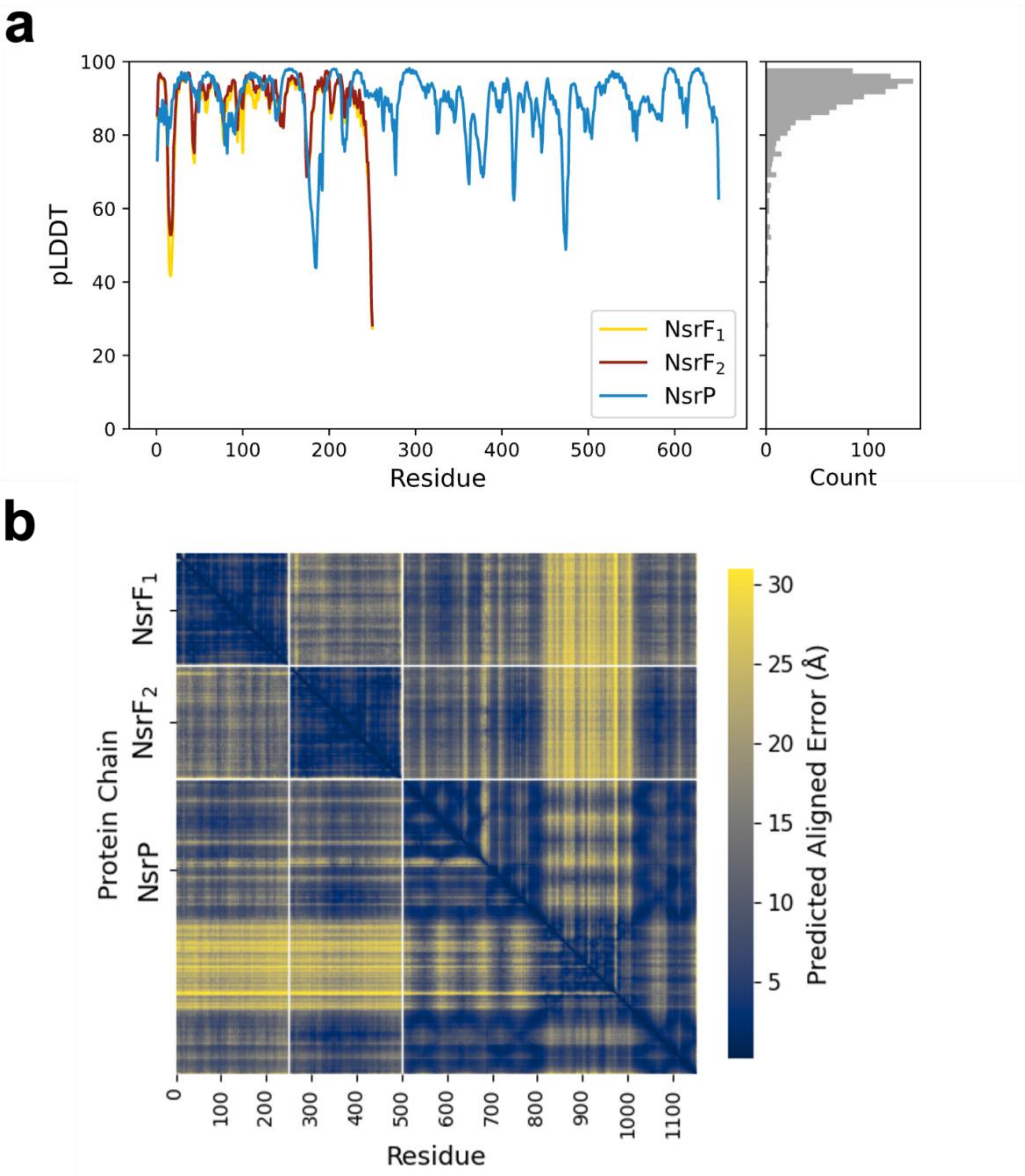

Supplementary Figure 5: *Sa*NsrFP model quality assessment. a. Predicted local distance difference test (pLDDT) for each protein chain of the model. The right panel shows a histogram with the overall score distribution. pLDDT values above 70 are considered a likely correct placement of the backbone atoms. b. Predicted aligned error for the model (PAE). The highest values observed correspond to the error associated with the relative orientation of the ECD with respect to the NBD domains.

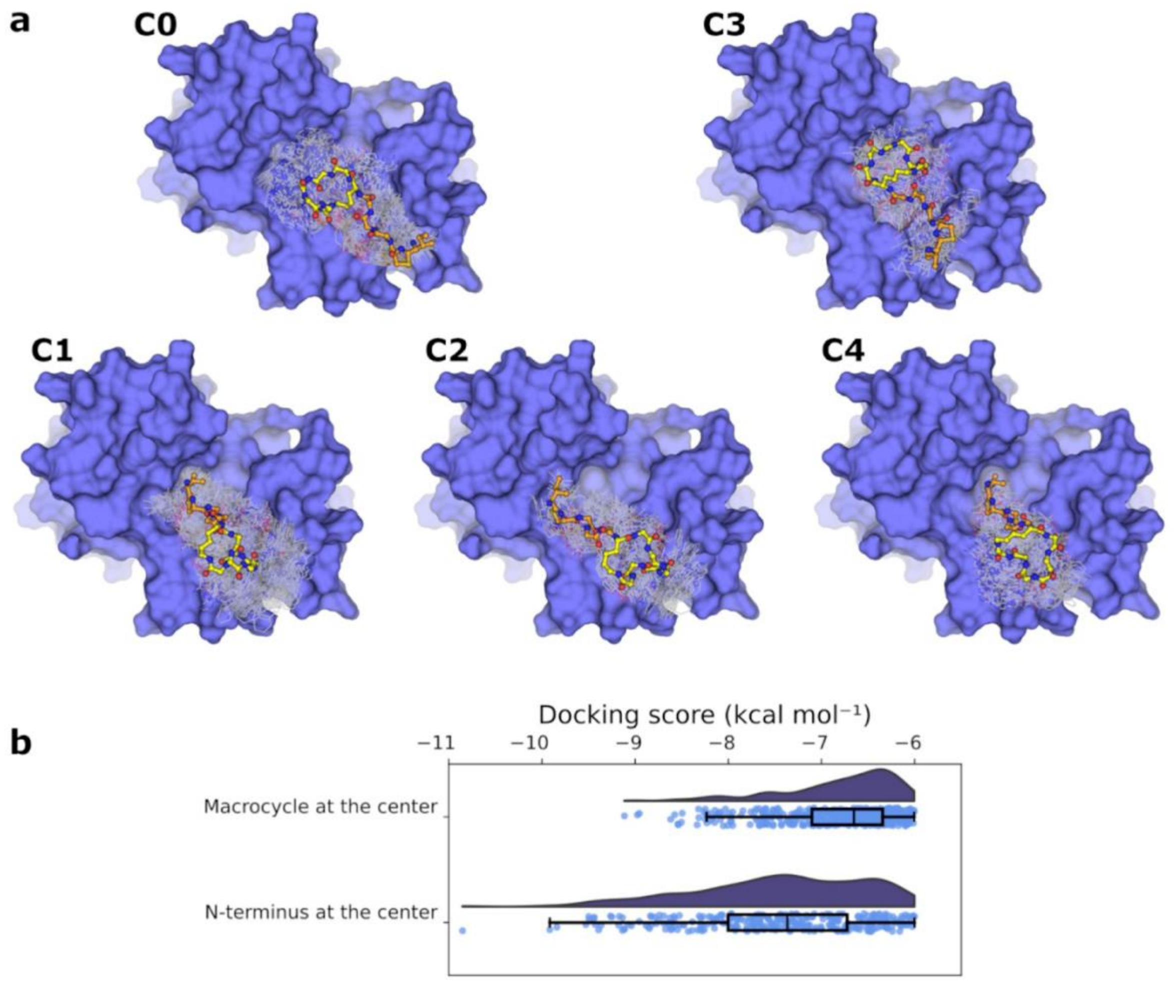

Supplementary Figure 6: Docking ensembles reveal two alternative configurations of bacitracin within the ECD. a) Top five most populated clusters resulting from docking (C0 to C4). For the best-scoring pose of the cluster, the backbone of the N-terminus is shown as orange sticks, whereas the macrocycle region is shown as yellow sticks All the other poses belonging to a cluster are shown as transparent white sticks. The ECD is shown as blue surface, with the portion connecting to the transmembrane domain placed at the bottom. The two clusters where the ring is located at the center of the ECD are shown at the top and the three clusters with the N terminus placed at the central cleft are shown at the bottom. b) Distributions of docking scores of the poses belonging to clusters where the macrocyclic region is at the center (C0 and C3) and clusters where the N-terminus is at the center (C1, C2, and C4). See Figure 1 for a description of the graph style. Both distributions are significantly different ($p = 1.02 \cdot 10^{-23}$, Mann-Whitney U test).

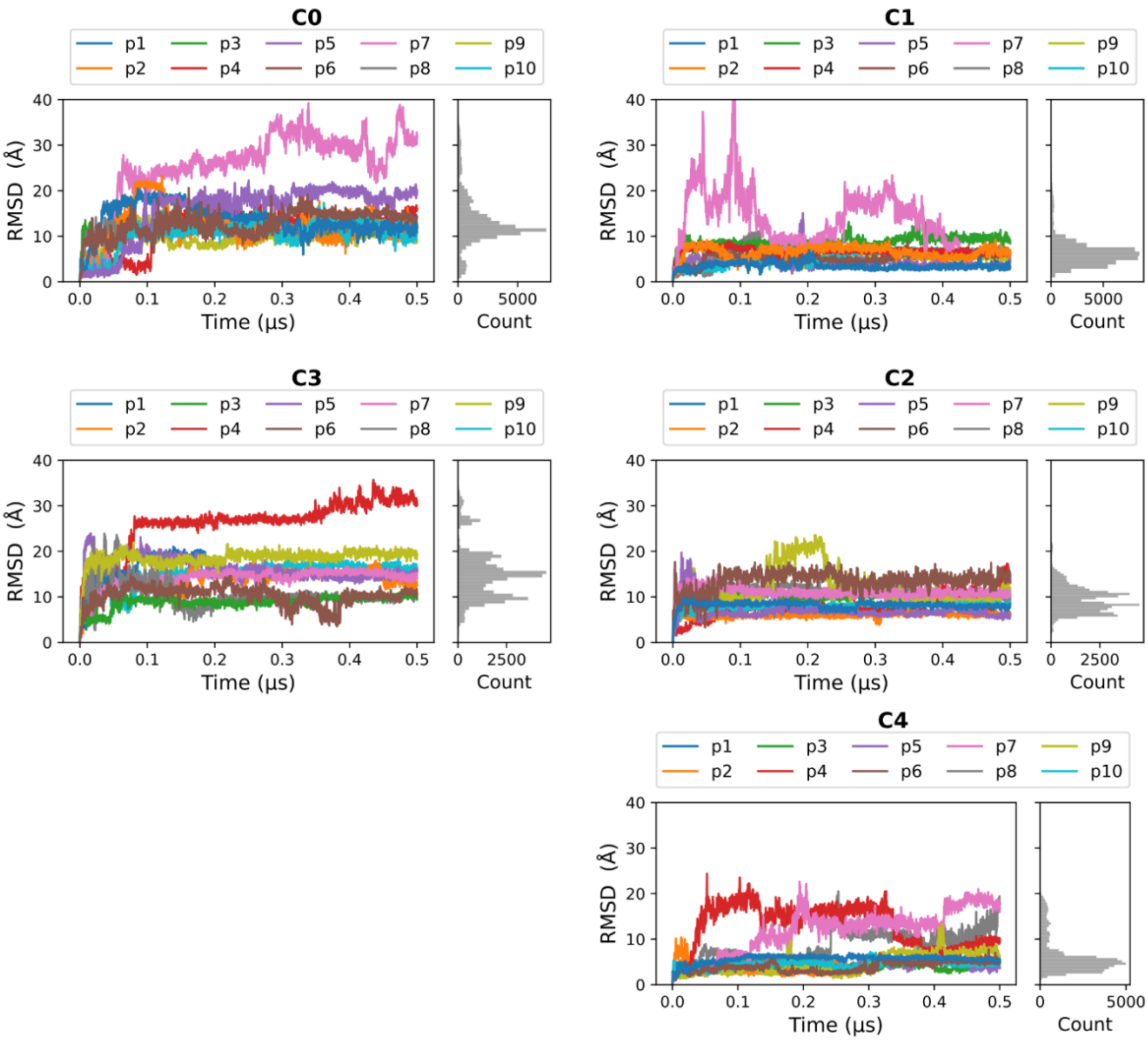

Supplementary Figure 7: RMSD of the center of mass of the macrocyclic ring of bacitracin for the best pose of each major docking solution cluster. Each replica is labeled as p*N*, where *N* is the replica number. A histogram showing the total RMSD distribution is shown at the right of each plot.

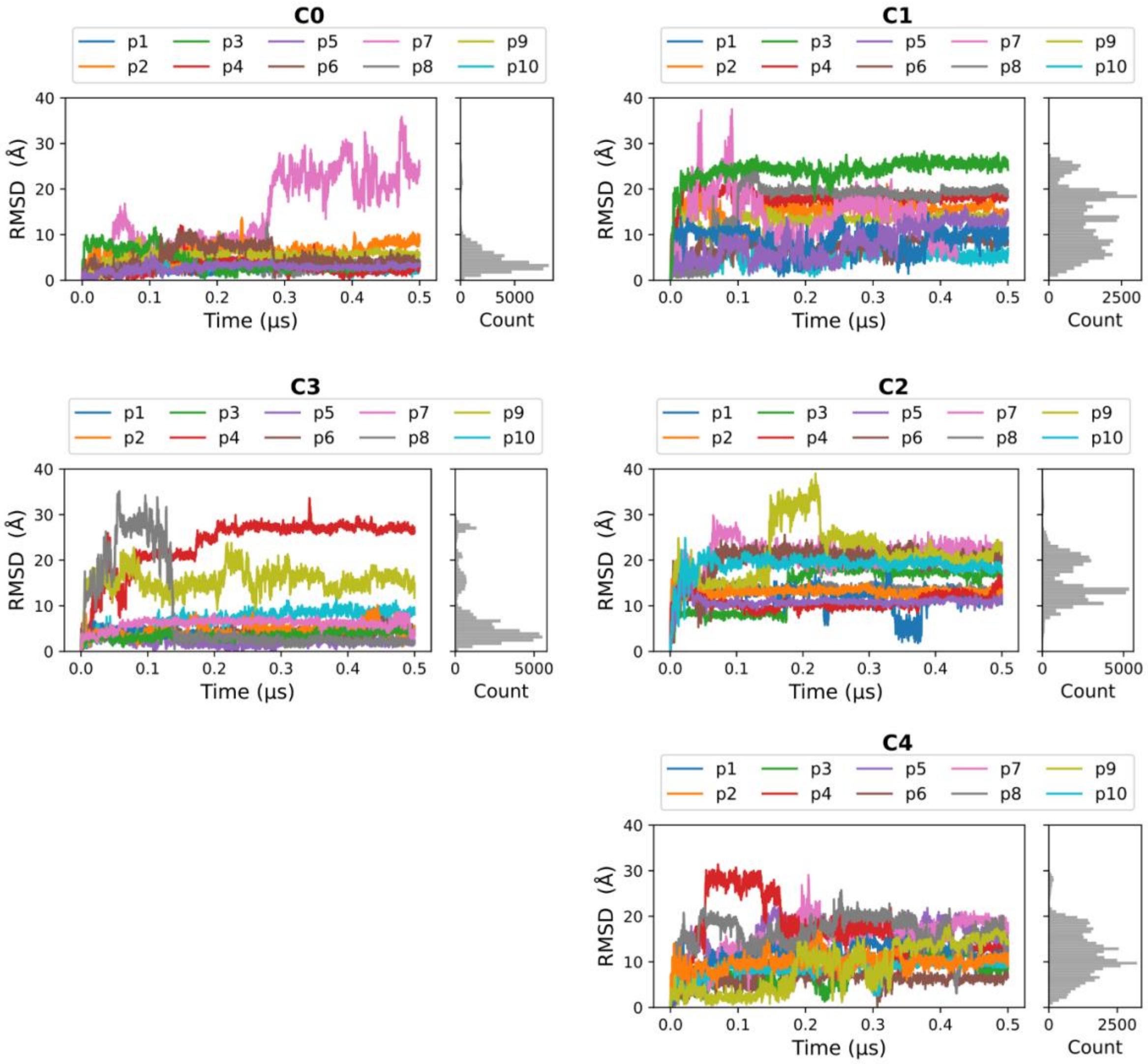

Supplementary Figure 8: RMSD of the backbone atoms of the N-terminal region of bacitracin for the best pose of each major docking solution cluster. Each replica is labeled as p*N*, where *N* is the replica number. A histogram showing the total RMSD distribution is shown at the right of each plot.

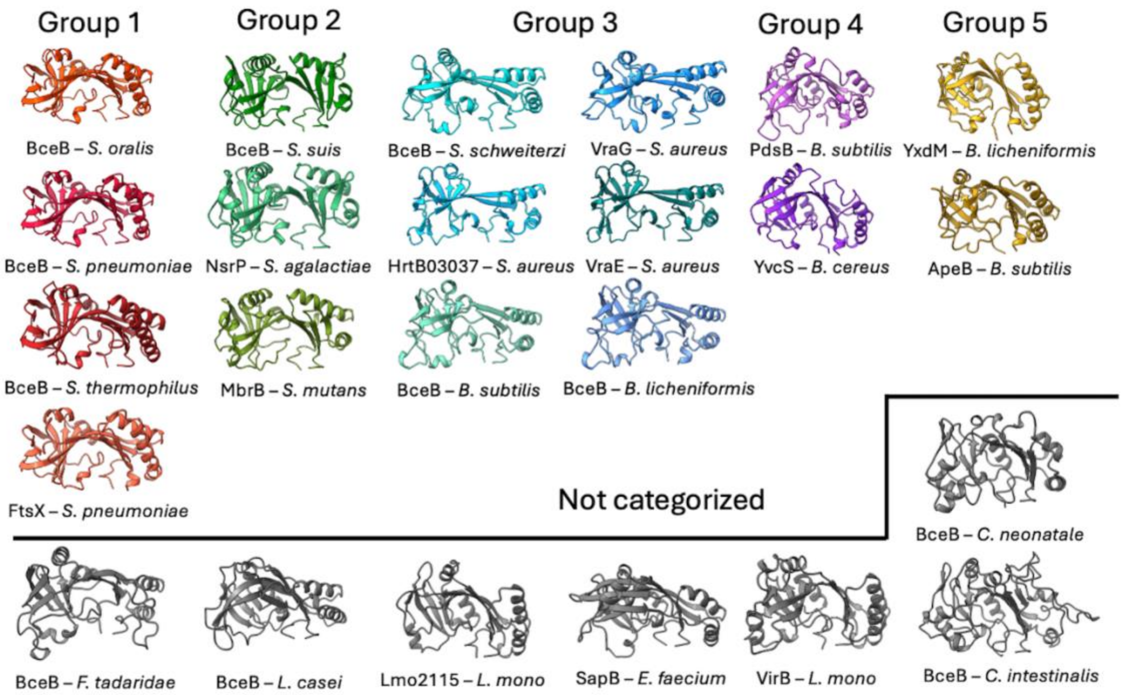

Supplementary Figure 9: Classification of all ECDs. Depicted are the AlphaFold models of all 24 ECDs and the classification into group 1 (red), 2 (green), 3 (blue), 4 (violet) and 5 (gold) based on an average RMSD < 2.5 Å. Additionally, the AlphaFold models of the uncategorized ECDs are shown in dark grey. Image was created with ChimeraX Version 1.8 and all accession numbers are stated in supplementary table 2.

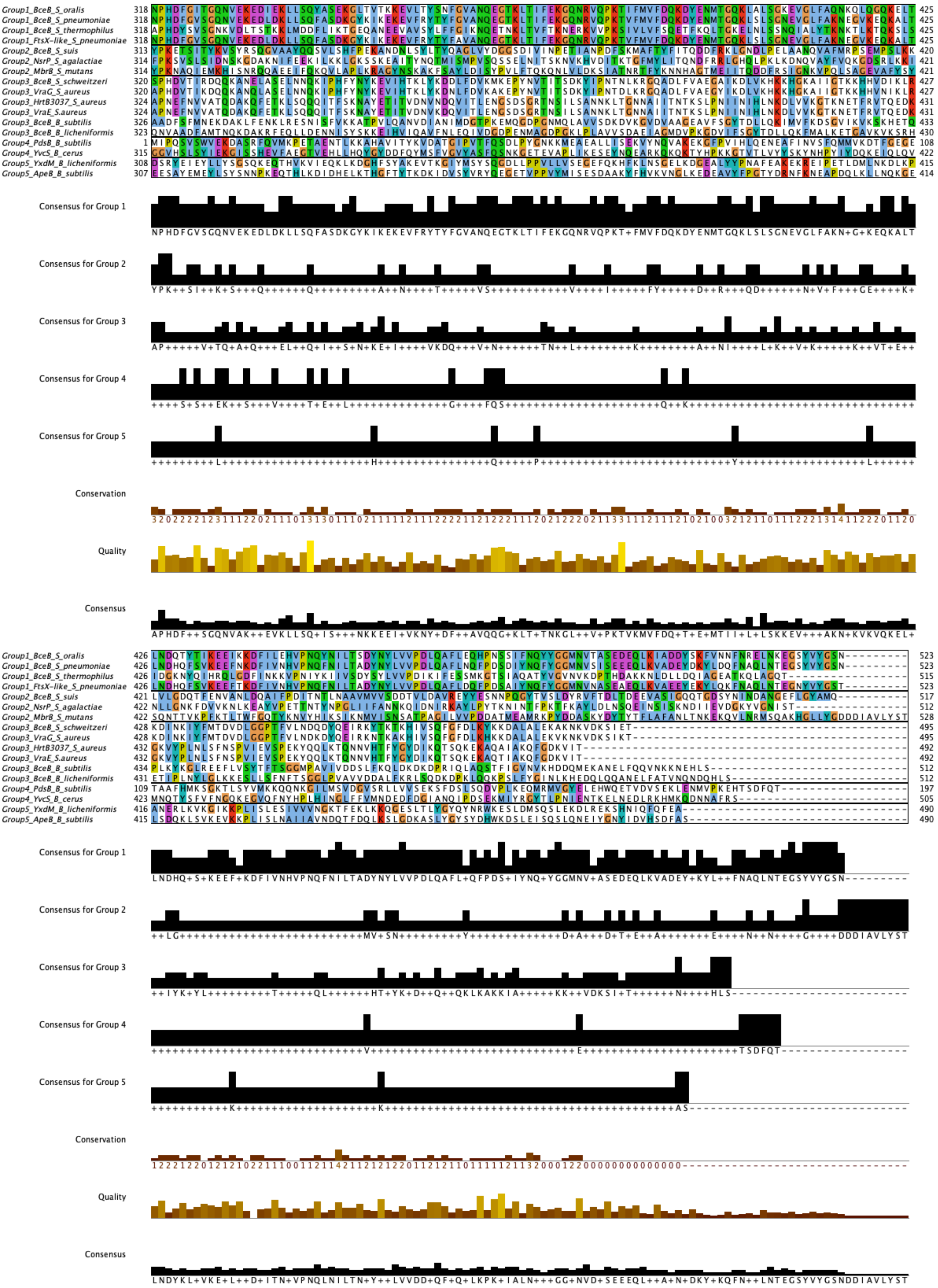

Supplementary Figure 10: Sequence alignment of all ECDs. ECD sequences were aligned with the Clustal Omega multiple sequence alignment server (Madeira et al., 2024).Conservation and consensus sequences are shown for each group. All accession numbers are stated in supplementary table 2.

Supplementary Table 2: All identified BceBs with related organism, grouping, Uniprot accession number, and AlphaFold identifier. Na.: not applicable.

Name	Organism	Group	Uniprot	AlphaFold
BceB	*Streptococcus oralis*	1	A0A3P5WUW9	AF-A0A3P5WUW9-F1-v4
BceB	*Streptococcus pneumoniae*	1	A0A4M3JMV1	AF-A0A4M3JMV1-F1-v4
BceB	*Streptococcus thermophilus*	1	A0A2X3UN27	AF-A0A2X3UN27-F1-v4
FtsX-like	*Streptococcus pneumoniae*	1	Q8DQ76	AF-Q8DQ76-F1-v4
BceB suis	*Streptococcus suis*	2	A0A0Z8AJY8	AF-A0A0Z8AJY8-F1-v4
NsrP	*Streptococcus agalactiae*	2	Q8DZX0	AF-Q8DZX0-F1-v4
MbrB	*Streptococcus mutans*	2	Q8VUH1	AF-Q8VUH1-F1-v4
BceB	Staphylococcus schweitzeri	3	A0A077UKU3	AF-A0A077UKU3-F1-v4
VraG	*Staphylococcus aureus*	3	Q9KWJ4	AF-Q9KWJ4-F1-v4
HrtB03037	*Staphylococcus aureus*	3	Q2FUR5	AF-Q2FUR5-F1-v4
VraE	*Staphylococcus aureus*	3	A0A0H3K209	AF-A0A0H3K209-F1-v4
BceB	*Bacillus subtilis*	3	O34741	AF-O34741-F1-v4
BceB	*Bacillus licheniformis*	3	Q65D41	AF-Q65D41-F1-v4
PdsB	*Bacillus subtilis*	4	A0A6M4JPG5	AF-A0A6M4JPG5-F1-v4
YvcS	*Bacillus cereus*	4	A0A164C1L6	AF-A0A164C1L6-F1-v4
YxdM	*Bacillus licheniformis*	5	A0A1Y0YLR7	AF-A0A1Y0YLR7-F1-v4
ApeB	*Bacillus subtilis*	5	P42424	AF-P42424-F1-v4
BceB	*Filibacter tadaridae*	Na.	A0A3P5WUW9	AF-A0A3P5WUW9-F1-v4
BceB	*Lactobacillus casei*	Na.	A0A6L3UJK5	AF-A0A6L3UJK5-F1-v4
Lmo2115	*Listeria monocytogenes serovar*	Na.	Q8Y5E9	AF-Q8Y5E9-F1-v4
SapB	*Enterococcus faecium*	Na.	R2P785	AF-R2P785-F1-v4
VirB	*Listeria monocytogenes serovar*	Na.	Q8Y6E1	AF-Q8Y6E1-F1-v4
BceB	*Collinsella intestinalis*	Na.	A0A5K1J584	AF-A0A5K1J584-F1-v4
BceB	*Clostridium neonatale*	Na.	A0A653APP4	AF-A0A653APP4-F1-v4

Supplementary Table 3: Primers used in this publication.

Name	Sequence
NsrFP_fw	5'- CATATCGAAGGTCGTCAT ATGTTATTAGAAATCAATC-3'
NsrFP_rv	5'- CCAAGGGGTTATGCTAG TTAGCGTTCAATAATATG-3'
H202A_fw	5'-GATGGTAACCGCTTCAGCAAATGCTG-3'
H202A_rv	5'-AAAATAGTTTGTCCATCCAAG-3'

References

KANONENBERG, K., ROYES, J., KEDROV, A., POSCHMANN, G., ANGIUS, F., SOLGADI, A., et al. 2019. Shaping the lipid composition of bacterial membranes for membrane protein production. *Microb Cell Fact,* 18**,** 131.

KIKHNEY, A. G., BORGES, C. R., MOLODENSKIY, D. S., JEFFRIES, C. M. & SVERGUN, D. I. 2020. SASBDB: Towards an automatically curated and validated repository for biological scattering data. *Protein Sci,* 29**,** 66-75.

KONAREV, P. V., VOLKOV, V. V., SOKOLOVA, A. V., KOCH, M. H. J. & SVERGUN, D. I. 2003. PRIMUS: a Windows PC-based system for small-angle scattering data analysis. *J Appl Crystallogr,* 36**,** 1277-1282.

MADEIRA, F., MADHUSOODANAN, N., LEE, J., EUSEBI, A., NIEWIELSKA, A., TIVEY, A. R. N., et al. 2024. The EMBL-EBI Job Dispatcher sequence analysis tools framework in 2024. *Nucleic acids res,* 52**,** W521-W525.

MANALASTAS-CANTOS, K., KONAREV, P. V., HAJIZADEH, N. R., KIKHNEY, A. G., PETOUKHOV, M. V., MOLODENSKIY, D. S.,et al. 2021. ATSAS 3.0: expanded functionality and new tools for small-angle scattering data analysis. *J Appl Crystallogr,* 54.

POROD, G. 1951. Die Röntgenkleinwinkelstreuung Von Dichtgepackten Kolloiden Systemen - 1 Teil. *Kolloid-Zeitschrift and Zeitschrift Fur Polymere,* 124**,** 83-114.

PYMOL 2022. The PyMOL Molecular Graphics System, Version 2.5 Schrödinger, LLC.

SVERGUN, D. I. 1992. Determination of the Regularization Parameter in Indirect-Transform Methods Using Perceptual Criteria. *J of Appl Crystallogr,* 25**,** 495-503.

SVERGUN, D. I., PETOUKHOV, M. V. & KOCH, M. H. 2001. Determination of domain structure of proteins from X-ray solution scattering. *Biophys J,* 80**,** 2946-53.

TULLY, M. D., KIEFFER, J., BRENNICH, M. E., COHEN ABERDAM, R., FLORIAL, J. B., HUTIN, S., et al. 2023. BioSAXS at European Synchrotron Radiation Facility - Extremely Brilliant Source: BM29 with an upgraded source, detector, robot, sample environment, data collection and analysis software. *J Synchrotron Radiat,* 30**,** 258-266.
